# Genetic testing of newborns for type 1 diabetes susceptibility: a prospective cohort study on effects on maternal mental health

**DOI:** 10.1186/1471-2350-11-112

**Published:** 2010-07-15

**Authors:** Kaja K Aas, Kristian Tambs, Marit S Kise, Per Magnus, Kjersti S Rønningen

**Affiliations:** 1 Division of Epidemiology, Norwegian Institute of Public Health, Oslo, Norway; 2 Division of Mental Health, Norwegian Institute of Public Health, Oslo, Norway; 3 Norwegian Directorate of Health, Oslo, Norway

## Abstract

**Background:**

Concerns about the general psychological impact of genetic testing have been raised. In the Environmental Triggers of Type 1 Diabetes (MIDIA) study, genetic testing was performed for HLA-conferred type 1 diabetes susceptibility among Norwegian newborns. The present study assessed whether mothers of children who test positively suffer from poorer mental health and well-being after receiving genetic risk information about their children.

**Methods:**

The study was based on questionnaire data from the Norwegian Mother and Child Cohort (MoBa) study conducted by the Norwegian Institute of Public Health. Many of the mothers in the MoBa study also took part in the MIDIA study, in which their newborn children were tested for HLA-conferred genetic susceptibility for type 1 diabetes. We used MoBa questionnaire data from the 30^th ^week of pregnancy (baseline) and 6 months post-partum (3-3.5 months after disclosure of test results). We measured maternal symptoms of anxiety and depression (SCL-8), maternal self-esteem (RSES), and satisfaction with life (SWLS). The mothers also reported whether they were seriously worried about their child 6 months post-partum. We compared questionnaire data from mothers who had received information about having a newborn with high genetic risk for type 1 diabetes (N = 166) with data from mothers who were informed that their baby did not have a high-risk genotype (N = 7224). The association between genetic risk information and maternal mental health was analysed using multiple linear regression analysis, controlling for baseline mental health scores.

**Results:**

Information on genetic risk in newborns was found to have no significant impact on maternal symptoms of anxiety and depression (*p *= 0.9), self-esteem (*p *= 0.2), satisfaction with life (*p *= 0.2), or serious worry about their child (OR = 0.98, 95% CI 0.64-1.48). Mental health before birth was strongly associated with mental health after birth. In addition, an increased risk of maternal worry was found if the mother herself had type 1 diabetes (OR = 2.39, 95% CI 1.2-4.78).

**Conclusions:**

This study did not find evidence supporting the notion that genetic risk information about newborns has a negative impact on the mental health of Norwegian mothers.

## Background

Predictive genetic testing is available for a range of diseases, from single gene diseases, such as Huntington's disease and cystic fibrosis, to multi-factorial diseases, such as type 1 diabetes, hereditary cancers, and familial hypercholesterolemia.

Type 1 diabetes is a complex disease for which important genetic components have been identified. A specific combination of the HLA class II genes (HLA-DRB1*0401-DQA1*03-DQB1*0302/DRB1*03-DQA1*05-DQB1*02) will give those who carry this genotype a 20% lifetime risk for developing type 1 diabetes [1-5]. In the Environmental Triggers of Type 1 Diabetes (MIDIA) study Norwegian newborns carrying this high-risk genotype were identified and followed in a quest to identify the environmental factors of the disease [6,7]. In the Norwegian population, 2.1% of newborns have this genotype, and this group represents approximately 34% of future cases of type 1 diabetes [5].

Several other studies have used predictive genetic testing of newborns as a strategy to solve research questions about environmental factors contributing to type 1 diabetes, including DIPP in Finland [8], PANDA in Florida [9,10], DiPiS in Sweden [11], DAISY in Colorado [12], and the multinational TEDDY study in the USA and Europe [13-15]. The main advantage for study participants identified as high-risk individuals is the possibility of early detection of the destruction of the insulin producing cells by autoantibodies, resulting in a milder disease onset [16], and the possibility of being the first to participate in intervention studies when possible preventive agents get available. However, there may be disadvantages of living with the knowledge of an increased susceptibility to a disease with no known prevention. Thus, even though predictive testing is highly acknowledged as a valuable research method per se, the predictive testing of children has given rise to concerned debate [17-19]. In an article about ethics, Ross concluded that, if the research does not incorporate a prevention strategy, studies involving predictive genetic testing of newborns should avoid disclosing test results to minimize the harm to infants and their families [18].

With the widespread and increasing use of genetic tests, assessing the adverse effects of information about susceptibility genes for disease on the tested subjects is important. Our study aimed to estimate the effect on maternal mental health from receiving genetic risk information about their newborns. Outcome measures were maternal self-report scores of anxiety and depression symptoms, satisfaction with life, self-esteem, and serious worry about the child. A number of previous studies [20-24] have examined maternal reactions after being informed that their children have an elevated genetic risk for type 1 diabetes. None of these studies have shown a significant effect on symptoms of anxiety or other mental health disorders as a result of the testing, though a few mothers did seem to react strongly. Previous studies were conducted in a setting in which the mothers were asked questions about how they felt in connection with the genetic testing project. The present study was designed differently. When completing the questionnaire, the mothers were not aware that their answers were going to be used for any particular comparisons, though they were rightfully informed that the personal data would be used for multiple research purposes. Thus, our results were not affected by reporting bias associated with maternal attitudes towards genetic risk information or other factors motivating the mothers to under- or over-report poor mental health.

In the present study, information about mental health is reported twice: before and after the mothers received the information about genetic risk. These data permit us to answer our main research question of to what extent receiving information about a young child having an increased risk of type 1 diabetes changes maternal well-being and mental health.

## Methods

### Study design

This study was based on questionnaire data from the Norwegian Mother and Child Cohort (MoBa) study [25,26], an ongoing longitudinal study of the general health and well-being of mothers and their children, starting during pregnancy. A subgroup of the mothers in the MoBa study participated in the MIDIA study (Environmental Triggers of Type 1 Diabetes) [6,7] as well. In MIDIA, the newborn child was tested for HLA-conferred genetic susceptibility to type 1 diabetes. Questionnaire data from the MoBa study were retrieved and used for the analysis of mental health measures for mothers whose children were genotyped in the MIDIA study. MoBa questionnaire data from the 30^th ^week of pregnancy ("the 30^th ^week questionnaire") and 6 months post-partum (3-3.5 months after disclosure of test results, "the 6 month questionnaire") were used for the analysis.

### Recruitment

The MoBa study was conducted by the Norwegian Institute of Public Health [26]. In brief, MoBa is a cohort consisting of more than 100 000 pregnancies recruited between 1999 and 2008. The majority of all pregnant women in Norway were invited to participate, and the participation rate was approximately 44%. Participants were recruited for the study by postal invitation in connection with being invited to a routine ultrasound examination offered to all pregnant women in Norway at 17-18 weeks of gestation http://www.fhi.no/morogbarn. Informed consent was obtained from each participant before inclusion in the study, which was approved by the Regional Committee for Medical Research and the Norwegian Data Inspectorate. The current study is based on version 3 of the quality-assured data files released for research in 2007. Information from the Medical Birth Registry of Norway (MBRN) is also included in the dataset [27].

The MIDIA study is a longitudinal prospective study in which newborns from the general Norwegian population carrying the high-risk HLA-DRB1*0401-DQA1*03-DQB1*0302/DRB1*03-DQA1*05-DQB1*02 genotype were identified and followed for the development of islet autoantibodies and the onset of type 1 diabetes [6,7]. Approximately 50% of the children tested in MIDIA have mothers who also participated in MoBa. Recruitment for the MIDIA study, which started in 2001, was described in detail by Stene *et al*. [7]. All health care nurses across Norway who took part in the recruitment, were trained to give correct information about the project. Informed consent was obtained from each participant before inclusion in the study, which was approved by the Regional Committee for Medical Research and the Norwegian Data Inspectorate.

### Study participants

The mothers included in this study delivered between 2001 and 2006, with the majority of deliveries in 2005 (37%). The study initially included 9762 mothers who had their child genotyped in MIDIA and, at the same time, participated in the MoBa study. Of these mothers, 7801 returned both the baseline (30^th ^week of gestation) and follow-up questionnaires (6 months post-partum). We excluded 411 pregnancies of women with more than one child. In most cases the first pregnancy or the first twin was included. In six cases the older sibling was excluded and the youngest included because the younger sibling was a high-risk child. A total of 7390 eligible participants were left for analysis.

### Procedure for communicating genotype results in the MIDIA study

#### Information to parents of children with the high-risk genotype

The high-risk genotype information was conveyed to the parents first by an initial phone call from one of four health care workers on the MIDIA project. The information provided during the phone call followed a semi-structural scheme to ensure that important topics were correctly communicated, including how to understand the test results. They were also told that the genes were inherited from both the mother and the father. The parents were informed that they would receive a letter a few days later with much of the same information in written form. In the letter, the risk was explained with the wording: "Children with the 'diabetes risk genes' have a 20% lifetime risk of developing diabetes (one of five) and a 7% risk of developing diabetes by the age of 15 years (1 of 17 children)." All parents were told that they were welcome to call the project if they had more questions or were worried about aspects related to the increased genetic risk. After 1-2 weeks, the parents received a second phone call with the purpose of clarifying unanswered questions and providing additional information if needed. In addition, all questionnaires received in the MIDIA study were scrutinized by one of the nurses to detect negative reactions due to the risk notification and to become aware of mothers or fathers who were thinking about their child's high genetic risk for type 1 diabetes daily. If such signs were found, the parents were contacted by phone. The term "diabetes risk genes" was used in all communication with the parents, whereas the perhaps more frightening term "high-risk genes" was avoided.

#### Information to parents of children without the high-risk genotype

The parents of children without the high-risk genotype were informed by letter. The letter stated that their child did not have the diabetes risk genes, but it also informed the parents that children without these genes can develop type 1 diabetes. These children were not offered further follow-up.

### Follow-up of children with high genetic risk in the MIDIA study

It is of relevance for the present study, how the children were followed-up between the high risk information was provided and until 6 months of age. Follow-up after 6 months is described in Stene *et al*. [7]. When the high-risk-children in the MIDIA study were 3 months old, their parents received a package with a questionnaire. The package also included equipment for a blood sample to be taken at the health care clinic, and equipment for stool samples to be collected from the diaper when the child was 3, 4 and 5 months old [7]. The parents were told that they would be notified if their child's blood samples showed elevated levels of islet autoantibodies. A similar package was received when the child was 6 months old. The 6 month follow-up package was sent to the parents approximately 2 weeks prior to the child turning 6 months old.

### Questionnaire variables

In the MoBa study, the questions concerning mental health were part of the questionnaire completed during the 17^th ^and 30^th ^week of pregnancy and when the child was 6 months, 18 months, and 3 years old. The present study used data from the 30^th ^week of pregnancy and 6 month questionnaires (Additional file 1 and Additional file 2). The 6 month questionnaire from the MoBa study was sent to the parents the same week as the child became 6 months old.

#### Maternal symptoms of anxiety and depression: SCL-8

Maternal symptoms of anxiety and depression were assessed using a short version of the SCL-25 [28]. The short version, SCL-8, consists of eight items: four items measuring depression and four items measuring anxiety. The correlation between SCL-8 and SCL-25 is 0.94 [29]. Shorter versions of SCL-25 have been found to perform almost as well as the full version when measuring anxiety and depression [30,31]. SCL-8 has been demonstrated to have an internal consistency of α = 0.88 [29].

A global SCL-8 score was calculated as a sum of the eight item scores. As the distribution of the total SCL-8 scores was positively skewed, a natural log transformation [ln(sum score)] was applied to the scores in order to approximate a normal distribution. The skewness was reduced from 2.6 to 1.2 and kurtosis from 10.3 to 1.2 for the SCL-8 variable at 6 months. The baseline SCL-8 variable was also transformed using the natural logarithm (ln). Cronbach's alpha (internal consistency) in the present sample was 0.83 for SCL-8.

#### The satisfaction with life scale

The five-item Satisfaction With Life Scale (SWLS) [32] was developed to measure the cognitive component of subjective well-being. In the original validation study [32], the SWLS demonstrated an internal consistency of α = 0.87. The instrument was calculated as a sum of the five individual item scores. Cronbach's alpha in the present data set was 0.88 for SWLS.

#### Rosenberg self-esteem scale

The short-form of the Rosenberg Self-Esteem Scale (RSES) [33] used in the MoBa study includes four items. These four items had a 0.95 correlation with the original ten item scale and an internal consistency of α = 0.80 [29]. The instrument was calculated as a sum of the four individual item scores (inverting the scores from the two middle questions). Cronbach's alpha in the present sample was 0.78 for RSES.

#### Maternal worry about the child

Maternal worry about the child was one of the items in an 11-item checklist of life events experienced during the last year, given in the 6 month questionnaire. The question was phrased 'Have you been seriously worried that there is something wrong with your child?' Responses were coded as 'yes' or 'no'.

#### Maternal diabetes status

A dichotomous variable was constructed to indicate the presence of maternal type 1 diabetes. The variable was based on health questions from both the MBRN [27] and the MoBa questionnaires. The mothers' own answers from the MoBa study were used to supplement and, if contradictory, correct the MBRN records. The agreement between questionnaire data and MBRN records was assessed as κ = 0.81. The MoBa questionnaire concerning father's health, including if he had type 1 diabetes or not, was not available for this study.

#### Sociodemographic characteristics of the participants

Sociodemographic characteristics from the MoBa questionnaires that were used to describe the sample were maternal income and maternal education. Maternal age at delivery and marital status were abstracted from the records of the MBRN.

### Child's genetic risk

A child's genetic risk was based directly on the results from the genotyping in the MIDIA study. The value of the variable was set to 1 if the mother had a newborn with high genetic risk for type 1 diabetes, and 0 if the mothers had a newborn without high genetic risk.

### Statistical analysis

#### Handling of missing data in SCL-8, RSES, and SWLS

The MVA (EM) imputation procedure in SPSS 14.0 was used to impute missing values for each construct item using the remaining construct items as predictors. Data from respondents with more than four values of SCL8 missing from the eight items in total were excluded from the analyses. Data from respondents with more than two values of SWLS missing from the five items in total were excluded from the analyses. Data from respondents with more than one value of RSES missing from the four items were excluded from the analyses.

The imputation of the SCL-8 items increased the sample with valid data from 6994 subjects (complete data on all eight SCL-8 items) to 7349 subjects at the 30^th ^week of pregnancy and from 6726 to 7361 subjects when the infant was 6 months old, whereas the number of subjects with valid data on both occasions increased from 6487 to 7321. Corresponding values for SWLS were 7277 subjects before and 7346 subjects after imputation (30^th ^week of pregnancy), 7237 before and 7308 after (when the infant was 6 months old), and 7134 before and 7266 after (valid data on both occasions). Valid RSES data increased from 7301 subjects to 7339 subjects (30^th ^week of pregnancy), from 7298 to 7345 (when the infant was 6 months old), and from 7215 to 7296 subjects (valid data on both occasions).

#### Regression analysis

Regression analysis included answers from mothers with valid data at both baseline (the 30^th ^week questionnaire) and after the disclosure of genetic risk (6 months questionnaire). Linear regression analyses were conducted separately for the dependent variables:

1. Symptoms of anxiety and depression (SCL-8)

2. Self-esteem (RSES)

3. Subjective well-being (SWLS).

In each of the three analyses, the dependent variable was the post-disclosure score for the maternal mental health variable. The independent variables were: child's genetic risk, maternal type 1 diabetes, and the baseline score for the maternal mental health variable. Exposure to risk information was the principal explanatory variable. By including the mental health baseline score as an independent variable, the effect of the independent variables can be interpreted as the effect in terms of a *change *in mental health from baseline to post-disclosure. Variables that are often chosen as covariates in multivariate analyses, such as demographic factors, are likely to be distributed by chance between the genetic high-risk and non-high-risk groups. As expected, preliminary bivariate tests showed no relationship between genetic risk and the demographic variables and, accordingly, demographic variables were not included in the analysis. However, maternal type 1 diabetes co-varies with the child's genetic risk and was entered as a covariate.

Logistic regression analysis was conducted with *maternal worry about the child *as a dependent variable. No baseline measurement was available for this dichotomous variable. *Child's genetic risk *and *maternal type 1 diabetes *were entered as predictors.

All analyses were conducted using SPSS, version 14.02.

## Results

### Subjects

There were no significant differences between the two study groups (Table 1) on the sociodemographic variables maternal education, marital status, maternal income, or maternal age (*p *> 0.27, chi-square tests). Thus, the sociodemographic variables were not included in the multivariate analyses.

**Table 1:** Sociodemographic characteristics of included study participants (N = 166 for high-risk group and N = 7224 for non-high-risk group)

	High-risk group			Non-high-risk group		
	No. of subjects	Percentage	95% CI	No. of subjects	Percentage	95% CI
	N*	%		N*	%	
**Maternal Education**						
Primary school (9 years)	0	0	0	132	1.8	1.5 - 2.1
Secondary school (10-13 years)	56	33.8	26.5 - 40.9	2236	30.9	29.9 - 32.0
College/University (≤ 4 years)	71	42.8	35.2 - 50.3	2984	41.3	40.2 - 42.5
College/University (> 4 years)	24	14.5	9.1 - 19.8	1464	20.3	19.3 - 21.2
						
**Marital status**						
Married	88	53	45.4 - 60.6	3703	51.3	50.1 - 52.4
Living together	67	40.4	32.9 - 47.8	3290	45.5	44.4 - 46.7
Divorced/Single/Other	5	3	0.4 - 5.6	162	2.2	1.9 - 2.6
						
Maternal income**						
< 200.000 NOK	51	30.8	23.7 - 37.7	1888	26.2	25.1 - 27.2
200.000-300.000 NOK	56	33.7	26.5 - 40.9	2683	37.1	36.0 - 38.3
300.000-400.000 NOK	37	22.3	16 - 28.6	1627	22.5	21.6 - 23.5
> 400.000 NOK	10	6	2.4 - 9.6	783	10.9	10.1 - 11.6
						
**Maternal age at delivery**						
< 25 years	15	9	4.7 - 13.4	648	9	8.3 - 9.6
25-34 years	121	72.9	66.1 - 79.7	5268	72.9	71.9 - 73.9
> 34 years	27	16.3	10.7 - 21.9	1265	17.5	16.6 - 18.4
						
**Maternal type 1 diabetes**	1	0.6	0 - 1. 8	36	0.5	0.3 - 0.7

* Because of missing data, not all numbers fits with the total number of participants ** NOK = Norwegian Krone

Comparisons with National data indicate that married or cohabitating mothers were over-represented in the study cohort (participants: 96.7%; national data: 89.6%) [34]. Furthermore, women with a college or university education were over-represented in the cohort (participants: 61.1%; national data: 36.1%) [35]. National data indicate that the mean maternal age when giving birth is 30.2 years [36], which is only slightly different from the present study group (mean 30.4, SD 4.4).

### Effects of genetic risk information on maternal mental health measures

The results from the linear regression analyses of the association between genetic risk information and change in maternal mental health are shown as unstandardized (B) and standardized (*β*) coefficients in Table 2. The upper part of Table 2 shows the results from the regression analysis with *symptoms of anxiety and depression *(SCL) as the dependent variable. The estimated regression coefficient (B = -0.001, *p *= 0.95) for *child's genetic risk *indicated no effect of genetic information on changes in maternal mental health from baseline to post-disclosure. The *maternal type 1 diabetes status *also had no effect (B = 0.040, *p *= 0.409). However, as expected, the *baseline anxiety/depression *score was strongly associated with post-disclosure scores (B = 0.536, *p *< 0.001).

**Table 2:** Effects of maternal diabetes and genetic risk information on mental health variables

	B (95% CI)	*β*	*p*	**Adjusted R**^**2**^
**Symptoms of anxiety and depression**				
Child's genetic risk	-0.001 (-0.047 - 0.044)	-0.001	0.953	
Maternal type 1 diabetes	0.040 (-0.055 - 0.135)	0.008	0.409	
Baseline anxiety/depression	0.536 (0.517 - 0.555)	0.544	< 0.001	
				0.296
**Self esteem**				
Child's genetic risk	0.037 (-0.022 - 0.097)	0.011	0.218	
Maternal type 1 diabetes	-0.040 (-0.164 - 0.083)	-0.006	0.521	
Baseline self esteem	0.682 (0.664 - 0.700)	0.651	< 0.001	
				0.423
**Satisfaction with life scale**				
Child's genetic risk	-0.080 (-0.198 - 0.039)	-0.013	0.187	
Maternal type 1 diabetes	0.016 (-0.231 - 0.263)	0.001	0.902	
Baseline SWLS	0.609 (0.590 - 0.628)	0.587	< 0.001	
				0.345

The middle and the lower part of Table 2 show similar negative results for the analysis of RSES and SWLS scores.

### Effects of genetic risk information on maternal worry about the child

Logistic regression was conducted with the mother's report on whether she felt seriously worried about her child (0 = no, 1 = yes) as a dependent variable. Maternal worry was measured in the 6 months questionnaire. Of the 7309 valid answers (excluding 81 participants who did not answer this question), 1227 mothers had felt serious worry about their child. Logistic regression revealed no difference between the mothers of high-risk and non-high-risk children (OR = 0.98, 95% CI 0.64-1.48, *p *= 0.91). An increased risk of maternal worry was seen if the mother herself had type 1 diabetes (OR = 2.39, 95% CI 1.20-4.78, p = 0.01).

## Discussion

This study compared mental health measures between mothers who received genetic risk information about their child and a control group. The results show no association between maternal notification of the results from predictive genetic testing of newborns for type 1 diabetes and maternal symptoms of anxiety and depression, satisfaction with life, self-esteem, or serious worry about the child.

Our study design is strong in some respects. The participants were from the MoBa study, a national prospective pregnancy cohort in which some of the mothers also participated in genetic testing of their newborns for HLA-conferred susceptibility for type 1 diabetes as part of the MIDIA study. Unlike most studies on the effect of testing newborns for a genetic risk of type 1 diabetes [20-24], the responses were given in a "neutral context": that is, the MoBa study did not have a specific purpose of studying the effects of genetic risk, so the mothers were not prompted to mentally focus on what they were supposed to report or what the researchers might expect them to report about the effects of such painful information. The study sample consisted of 166 mothers of children with the high-risk genotype and a control group of 7224 mothers of children not carrying the high-risk genotype for type 1 diabetes. These large sample sizes provide adequate power for detecting differences between the two groups of mothers. The mental health measurements were reported both before and after receiving the information about genetic risk, providing the opportunity to adjust for baseline measurements in the analysis. Finally, consistency of the findings across three different validated measures of mental health strengthens the conclusion that mental health was not reduced by communicating genetic risk.

Previous studies examining the psychological reactions of mothers of children with high genetic risk for type 1 diabetes found no significant effect on anxiety or other serious mental health symptoms in the group as a whole in response to genetic risk information [20-24]. The present study confirmed the main results from previous studies. The contribution from our study is associated with an absence of context effects, large sample sizes, and available baseline scores. Weaknesses in previous studies were small sample sizes [22,24], lack of baseline measures [20,21,23], or no control group [20,21]. In some previous studies, effects of genetic risk information were revealed when the mothers were asked questions relating directly to the test results [22,23]. One week after obtaining the risk information, 55% of the mothers surveyed by Simonen *et al*. expressed modest worry about the test results [23]. In addition, Kerruish *et al*. reported higher levels of concern about the child's genetic risk status among mothers of high-risk children compared to low-risk children [22]. Because the questions in the present study were taken from questionnaires intended to study health and well-being in general, specific questions about concern about the genetic test results were not included, and therefore cannot be compared with these previous studies.

A weakness of the present study is the time lag between exposure to the risk information and observation of mental health. The MoBa 6 month questionnaire was distributed 3 to 3.5 months after information about risk was provided. Review studies investigating the effects of predictive genetic testing on adults have indicated that, over time, risk perception decreases [37], and test results were rarely predictive of distress more than one month after testing [38]. On the other hand, the MIDIA follow-up package, which is an obvious reminder about the child's risk, is sent to the parents around 2 weeks before completing the 6 months questionnaire. Overall, one cannot exclude that a difference between the mothers of high-risk and non-high-risk children would have been detected if maternal psychological health had been assessed closer to the time that the risk information was provided.

The parents of high-risk and non-high-risk children received risk information and counselling to a different extent. During the period after testing, the children with type 1 diabetes were subject to follow-up, including the opportunity for the parents to consult the project physician and health nurses at any time. The follow-up also included biological monitoring of blood samples to measure autoantibody levels, thus increasing the possibility of early diagnosis and a less severe onset of the disease if the child were to develop type 1 diabetes. If the mothers of high-risk children had been left by themselves after receiving the risk information, we cannot exclude some deterioration of maternal mental health.

Information about a child's seroconvertion is expected to lead to increased anxiety in the mothers, as this is a strong indicator of increased risk for development of type 1 diabetes [39]. However, none of the children seroconverted at the 3 months follow-up.

The findings of this study are not necessarily fully valid for genetic screening. In screening, all newborns would be enrolled for genetic testing, whereas in a prospective cohort study, participants decide, after careful consideration, if they want to participate in the study and receive the results. This may have led to self-selection of individuals who believe themselves to be fit to handle risk information. This assumption was supported in a recent study [40], which found that adults who anticipated having adverse psychological reactions to genetic testing were less likely to choose genetic testing. Likewise, Codori *et al*. compared a group who had chosen predictive testing for Huntington's disease and a group who had chosen not to be tested [41]. The conclusion was that the tested group anticipated problems with emotional reactions less often and expected to be able to cope with such reactions should they appear. Furthermore, Decruyenaere *et al*. found that the individuals who tested themselves for Huntington's disease had higher ego strength and were more socially extroverted than the general population [42].

One of the limitations of quantitative studies such as ours is the inability to acknowledge and be fully aware of "single case destinies", small number of mothers who might react very strongly to genetic risk information about their child. Although the present study does not show an association between predictive genetic testing and maternal mental health, previous studies have shown that such information could be a burden to a few individuals [20,21,23]. Hood *et al*. found that although depressive symptom scores are not elevated for the group as a whole, there is considerable variety in the responses [20]. Mothers with ethnic minority status, low education, and post-partum depression seem to be more likely to respond to risk notification with depressive symptoms. Similarly, Johnson *et al*. found that ethnic minority status, low education, and not being married are associated with elevated anxiety levels [21].

Mothers who have type 1 diabetes seemed to worry more about their child. The mothers from the group with not-high-risk children who expressed worry would be responding to something other than the gene test. The mothers could still worry about their child getting the disease, or another chronic disease.

## Conclusions

This study did not find evidence supporting the notion that genetic risk information about newborns has a negative impact on the mental health of Norwegian mothers.

## Competing interests

The authors declare that they have no competing interests.

## Authors' contributions

KKA participated in the data acquisition, performed the analyses and drafted the manuscript. KT supervised the analyses and helped to draft the manuscript. MSK participated in the data acquisition and helped to draft the manuscript. PM participated in the design, is the principal investigator for MoBa and helped to draft the manuscript. KSR conceived of the study, participated in the design, data acquisition and coordination and helped to draft the manuscript. All authors read and approved the final manuscript.

## Pre-publication history

The pre-publication history for this paper can be accessed here:

http://www.biomedcentral.com/1471-2350/11/112/prepub

## Supplementary Material

Additional file 1 **30^th ^week questionnaire**. The MoBa questionnaire from 30^th ^week of gestation. The Satisfaction With Life Scale (SWLS) corresponds to item no. 126, Rosenberg Self-Esteem Scale (RSES) to item 127, and Symptoms Check-List (SCL-8) to item 123. Click here for file

Additional file 2 **6 month questionnaire**. The MoBa questionnaire when the child is 6 months old. SWLS corresponds to item no. 90, "Maternal worry about the child" to item 91, RSES to item 93, and SCL-8 to item 94. Click here for file
