# Supplementary material for: Genetic testing of newborns for type 1 diabetes susceptibility: a prospective cohort study on effects on maternal mental health
Source: BMC Med Genet. 2010 Jul 15;11:112. doi: 10.1186/1471-2350-11-112 (PMC3152763; doi:10.1186/1471-2350-11-112)
Supplement: Additional file 1 — 30th week questionnaire. The MoBa questionnaire from 30th week of gestation. The Satisfaction With Life Scale (SWLS) corresponds to item no. 126, Rosenberg Self-Esteem Scale (RSES) to item 127, and Symptoms Check-List (SCL-8) to item 123. [file 1471-2350-11-112-S1.PDF]

den norske **Mor & barn undersøkelsen**

Questionnaire 3C

This questionnaire applies mainly to the period after week 12 of your pregnancy. We will ask you some questions which you may recognise from the first questionnaire. We do this because we want to continue following your and your child's progress. It would be useful for you to consult your pregnancy health card before you start answering the questions so that you can use the information contained in it when completing this questionnaire. If you feel uncomfortable with a question or it is difficult to answer, you can skip this question and go on to the next one.

This questionnaire will be processed by a computer. It is therefore important that you follow these instructions:

- Use a blue or black ballpoint pen.
- Put a cross in the box that is most relevant like this: ☒
- If you put a cross in the wrong box, correct it by filling in the box completely like this: ■
- Write a number or capital letter in the large green boxes.

It is important that you only write in the white area of each box like this:

Number: 1 2 3 4 5 6 7 8 9 0 Letter: A B C D

- When entering a single-digit number in boxes containing two or more squares, use the square on the right. **For example:** 5 is written like this: ☐ 5
- A number of questions in this questionnaire concern the week of pregnancy. *For example: If you want to indicate something that happened 14 weeks after your last period, enter a cross in the box for week 13-16.*
- Specific information concerning, for example, medication or profession should be written in the boxes or on the lines provided. Please write clearly in CAPITAL LETTERS.
- Remember to enter the date when you completed the questionnaire.

Please return the completed questionnaire in the stamped addressed envelope provided.

Date when the questionnaire was completed ☐☐ Day ☐☐ Month ☐☐☐☐ Year (write the year in full, e.g. 2001)

Antenatal care and health

1. Where have you been to antenatal check- ups? (Fill in one or more boxes.) Specify how many times.

- ☐ Public health centre ☐☐ times
- ☐ Doctor's surgery ☐☐ times
- ☐ Hospital (outpatients) clinic ☐☐ times

2. Who has examined you each time? (Fill in one or more boxes.) Specify how many times.

- ☐ Midwife ☐☐ times
- ☐ General practitioner ☐☐ times
- ☐ Gynaecologist ☐☐ times
- ☐ Public health nurse ☐☐ times

3. Is your doctor male or female? How many times have you gone to him/her?

General practitioner	☐ female	☐☐ times
	☐ male	☐☐ times
Gynaecologist	☐ female	☐☐ times
	☐ male	☐☐ times

4. If you visit or have visited a gynaecologist or hospital clinic for your antenatal check-ups, what is or was the reason?

- ☐ Referred due to complications during this pregnancy
- ☐ Referred due to previous illness or complications in previous pregnancies
- ☐ On your own initiative without a referral
- ☐ Referred for another reason

Questionnaire 3c M&B 20,000 1101

5. Do you agree with the following statements concerning your antenatal check-ups?

	Agree completely	Agree	Agree somewhat	Disagree somewhat	Disagree	Disagree completely
I have been given sufficient advice and information	☐	☐	☐	☐	☐	☐
I have been well taken care of	☐	☐	☐	☐	☐	☐
There was not enough time during the consultations ..	☐	☐	☐	☐	☐	☐
I felt secure during these check-ups	☐	☐	☐	☐	☐	☐
I have been able to discuss everything I needed to during the check-ups	☐	☐	☐	☐	☐	☐
On the whole, I am satisfied with the way I have been followed up by the health service	☐	☐	☐	☐	☐	☐

6. Have you contacted a midwife or doctor in addition to your normal check-ups?

	No	Yes
Midwife................	☐	☐
Doctor	☐	☐

7.If yes, was it difficult to get an appointment?

	Midwife	Doctor
Not difficult	☐	☐
Somewhat difficult	☐	☐
Very difficult	☐	☐

8. Have you had a gynaecological examination during your pregnancy (internal examination)? If so, how many times?

☐ No
☐ Yes ☐☐ Times

9. How many ultrasound examinations have you had during your pregnancy?

External ultrasound examination ☐☐ Times

Internal ultrasound examination ☐☐ Times

10. How many children are you expecting? ☐

11. Have you been offered an amniocentesis or placenta biopsy?

☐ No (go to question 16)
☐ Yes

12. If yes, were any tests performed and what were the results?

	Was the test performed? Yes	No	Were the results normal? Yes	No
Amniocentesis	☐	☐	☐	☐
Placenta biopsy	☐	☐	☐	☐

If the tests were abnormal, describe the findings:

____

13. If an amniocentesis or placenta biopsy was performed, what was the reason?

☐ Due to my age (normally 38 or older at the time of delivery)
☐ Previous child with a chromosome disorder
☐ Previous child with neural tube defect (spina bifida)
☐ Epilepsy (medication for epilepsy)
☐ Ultrasound findings
☐ Other

14. Were there complications during the first 2 weeks following the amniocentesis?

☐ No
☐ Yes

15. If yes, what kind of complications?

☐ Vaginal bleeding
☐ Leakage of amniotic fluid
☐ Abdominal pain (similar to or stronger than menstrual pains)
☐ Other ______________________________

16. Have you had an X-ray during pregnancy?

☐ No
☐ Yes

17. If yes, what part of your body was X-rayed? How many X-rays were taken and in which week of pregnancy? (Fill in one or more boxes.)

	Week of pregnancy 0-12	13-16	17-20	21-24	25-28	29+	No. of times
Teeth	☐	☐	☐	☐	☐	☐	☐
Lungs.	☐	☐	☐	☐	☐	☐	☐
Arms or legs	☐	☐	☐	☐	☐	☐	☐
Pelvis/abdomen/ back	☐	☐	☐	☐	☐	☐	☐
Other	☐	☐	☐	☐	☐	☐	☐

18. Have you received treatment to prevent a premature birth during this pregnancy? (Fill in one or more boxes.)

☐ No
☐ Yes, relax or bed-rest
☐ Yes, medication

Which medicines? ______________________________

19. Have you been vaccinated during this pregnancy?

☐ No
☐ Yes

Which vaccine? ______________________________

20. Has the midwife or doctor told you that you have or have had high blood pressure during this pregnancy?

☐ No
☐ Yes

21. If yes, what was the highest reading during this pregnancy? (High blood pressure is over 140/90) (Refer to your health card.)

☐☐☐ / ☐☐☐ *E.g.* 150/ 95

☐ Don't know

22. Have you had high blood pressure without being pregnant?

☐ No
☐ Yes
☐ Don't know

23. If yes, what was the highest reading before this pregnancy?

☐☐☐ / ☐☐☐ *E.g.* 150/ 95

☐ Don't know

24. What was your blood percentage/haemoglobin (Hb) value during this pregnancy? (Refer to your health card and note the most recent, in addition to the highest and lowest values.)

	Haemoglobin (Hb)	Week of preg
Value at last antenatal check-up during pregnancy	☐☐,☐	☐☐
Highest value during pregnancy	☐☐,☐	☐☐
Lowest value during pregnancy	☐☐,☐	☐☐

☐ Don't know

25. How much did you weigh at your last antenatal check-up and when was it? (Refer to your health card.)

Weight ☐☐☐,☐ kg

Date of antenatal check -up ☐☐ ☐☐ ☐☐☐☐

Day Month Year

26. Have you been admitted to the hospital since you became pregnant?

☐ No

☐ Yes, which hospital(s) ____________________

27. If yes, why and when were you hospitalised? (Fill in one or more boxes.)

In which week of pregnancy were you admitted?

	0–4	5–8	9–12	13–16	17–20	21–24	25–28	29+
☐ Prolonged nausea and vomiting	☐	☐	☐	☐	☐	☐	☐	☐
☐ Bleeding	☐	☐	☐	☐	☐	☐	☐	☐
☐ Leakage of amniotic fluid	☐	☐	☐	☐	☐	☐	☐	☐
☐ Threatening preterm labour	☐	☐	☐	☐	☐	☐	☐	☐
☐ High blood pressure	☐	☐	☐	☐	☐	☐	☐	☐
☐ (Pre-)Eclampsia	☐	☐	☐	☐	☐	☐	☐	☐
☐ Other ____________________	☐	☐	☐	☐	☐	☐	☐	☐

28. Do you have or have you ever had any of the following?

			If yes, how often have you had problems?				How much at a time?	
**Before this pregnancy:**	No	Yes	1–4 times a month	1–6 times a week	Once a day	More than once a day	Drops	Large amounts
Incontinence when coughing, sneezing or laughing	☐	☐	☐	☐	☐	☐	☐	☐
Incontinence during physical activity (running / jumping)	☐	☐	☐	☐	☐	☐	☐	☐
Incontinence with a strong need to urinate	☐	☐	☐	☐	☐	☐	☐	☐
Problems retaining faeces	☐	☐	☐	☐	☐	☐	☐	☐
Problems with flatulence	☐	☐	☐	☐	☐	☐	☐	☐
**In this pregnancy:**								
Incontinence when coughing, sneezing or laughing.								
Incontinence during physical activity (running / jumping)	☐	☐	☐	☐	☐	☐	☐	☐
Incontinence with a strong need to urinate.	☐	☐	☐	☐	☐	☐	☐	☐
Problems retaining faeces	☐	☐	☐	☐	☐	☐	☐	☐
Problems with flatulence	☐	☐	☐	☐	☐	☐	☐	☐

29. Do you have or have you had pain in any of the following parts of your body? Where and when? (Fill in one or more boxes.)

		During this pregnancy		During earlier pregnancies		Between pregnancies		Prior to first pregnancy	
	No	Mild Pain	Severe Pain	Mild Pain	Severe Pain	Mild Pain	Severe Pain	Mild Pain	Severe Pain
Small of the back	☐	☐	☐	☐	☐	☐	☐	☐	☐
One of the pelvic/sacroiliac joints at the back	☐	☐	☐	☐	☐	☐	☐	☐	☐
Both pelvic/sacroiliac joints at the back	☐	☐	☐	☐	☐	☐	☐	☐	☐
Over the coccygeal bone	☐	☐	☐	☐	☐	☐	☐	☐	☐
In the buttocks	☐	☐	☐	☐	☐	☐	☐	☐	☐
Over the pubic bone	☐	☐	☐	☐	☐	☐	☐	☐	☐
Groin	☐	☐	☐	☐	☐	☐	☐	☐	☐
Other back pains	☐	☐	☐	☐	☐	☐	☐	☐	☐

30. Do you wake up at night due to pelvic pain?

- ☐ Yes, frequently
- ☐ Yes, sometimes
- ☐ No, never

31. Do you have to use a stick or crutches in order to walk due to pelvic pain?

- ☐ No, never
- ☐ Yes, but not every day, the pain varies from day to day
- ☐ Yes, I have to use a stick or crutches every day

32. Have you received an anaesthetic in connection with surgery or dental treatment during this pregnancy?

- ☐ No
- ☐ Yes

33. If yes, what type of anaesthetic have you had? (Fill in one or more boxes.)

- ☐ General (full) anaesthetic
- ☐ Spinal anaesthetic (epidural)
- ☐ Local anaesthetic
- ☐ Don't know

34. Have you been to the dentist during this pregnancy?

- ☐ No
- ☐ Yes

35. If yes, did the dentist perform any of the following treatments? (Fill in one or more boxes.)

	Yes	No
Put in new amalgam fillings (silver fillings)	☐	☐
Removed or replaced amalgam fillings	☐	☐
Put in new white fillings	☐	☐

36. How many teeth do you have and how many have fillings? (Look in the mirror and count.)

Total number of teeth ☐☐

Number of teeth with amalgam fillings ☐☐

Number of teeth with other types of fillings ☐☐

37. At present, do your gums bleed when you brush your teeth?

- ☐ No, seldom or never
- ☐ Yes, sometimes
- ☐ Yes, frequently
- ☐ Yes, nearly always

38. Have you had a tattoo or body piercing, including extra holes in the ears? (Do not include pierced ears if you have one hole in each ear.)

- ☐ No
- ☐ Yes

39. If yes, where and when was it done? (Fill in one or more boxes.)

	Tattoo	Body piercing
Before this pregnancy:		
In Norway	☐	☐
Abroad	☐	☐
During this pregnancy:		
In Norway	☐	☐
Abroad	☐	☐

40. Have you ever had a blood transfusion? If yes, give the number of transfusions.

- ☐ No
- ☐ Yes, during this pregnancy ☐☐ Times
- ☐ Yes, before this pregnancy ☐☐ Times

41. If yes, in which country and which year? (Give the last 2 transfusions.)

	YEAR
Country: ____________	☐☐☐☐
Country: ____________	☐☐☐☐

42. Have you ever had breast surgery?

- ☐ No
- ☐ Yes

43. If yes, was it:

- ☐ Breast enlargement
- ☐ Breast reduction
- ☐ Cancer/biopsy
- ☐ Other, describe: ____________

44. Have you ever had cervical dysplasia?

- ☐ No
- ☐ Yes

☐☐☐☐ Year the dysplasia was detected the first time

45. Have you had an operation on your cervix?

- ☐ No
- ☐ Yes

☐☐☐☐ Year of operation

46. Have you ever had a gamma globulin injection? (used to prevent infection of hepatitis A, primarily when travelling abroad.)

- ☐ No
- ☐ Yes

If yes, which year? ☐☐☐☐

How have you been recently?

Some questions about the time that has elapsed since the 13th week of pregnancy.

47. Have you had one or more episodes of vaginal bleeding after the 13th week of pregnancy?

☐ No
☐ Yes

48. If yes, how much did you bleed, in which week(s) of pregnancy and how many days did the bleeding last? (If you have had more than 2 episodes of bleeding, describe the last 2 only.)

	The amount of blood (spotting means a few drops)			In which week of pregnancy did the bleeding occur? 13–16	17–20	21–24	25–28	29+	No. of days bleeding lasted
1.	☐ Spotting	☐ More than spotting	☐ Large amounts	☐	☐	☐	☐	☐	☐☐
2.	☐ Spotting	☐ More than spotting	☐ Large amounts	☐	☐	☐	☐	☐	☐☐

☐ Number of episodes of bleeding if more than 2 ☐☐

49. Do you know why you bled?

☐ No
☐ Yes

50. If yes, what was the reason? (Fill in one or more boxes.)

☐ The placenta is too low/is in a difficult position/placenta previa
☐ Premature separation of the placenta/abruptio/ablatio placenta
☐ Threatening miscarriage/premature birth
☐ Cervical ulcer, bleeding of the mucous membrane in the vagina
☐ Following intercourse
☐ Other reason

51. Have you been bothered by uterine contractions?

☐ No
☐ Yes, a little
☐ Yes, a lot

52. Do you have or have you had any of the following illnesses or problems after the 13th week of pregnancy? If you have used tablets, mixtures, suppositories, inhalers, creams, etc. in connection with the illness or problem, give the name(s) of the medication(s), when and how long you took them. (Fill in one or more boxes.) (This applies to all types of medicines including alternative and herbal remedies, both regular and occasional use. Do not include vitamins and nutritional supplements as these are asked about elsewhere.)

	In which week of pregnancy did you have problems? 13-16	17-20	21-24	25-28	29+	The name of the medication taken	In which week of pregnancy did you take medication 13-16	17-20	21-24	25-28	29+	No. of days taken
1 Pelvic girdle pain . . .	☐	☐	☐	☐	☐	________	☐	☐	☐	☐	☐	☐☐☐
2 Back pains	☐	☐	☐	☐	☐	________	☐	☐	☐	☐	☐	☐☐☐
3 Other pains in muscles/joints	☐	☐	☐	☐	☐	________	☐	☐	☐	☐	☐	☐☐☐
4 Nausea	☐	☐	☐	☐	☐	________	☐	☐	☐	☐	☐	☐☐☐
5 Long-term nausea and vomiting	☐	☐	☐	☐	☐	________	☐	☐	☐	☐	☐	☐☐☐
6 Vaginal thrush	☐	☐	☐	☐	☐	________	☐	☐	☐	☐	☐	☐☐☐
7 Vaginal catarrh, unusual discharge . . .	☐	☐	☐	☐	☐	________	☐	☐	☐	☐	☐	☐☐☐
8 Pregnancy itch	☐	☐	☐	☐	☐	________	☐	☐	☐	☐	☐	☐☐☐
9 Constipation	☐	☐	☐	☐	☐	________	☐	☐	☐	☐	☐	☐☐☐
10 Diarrhoea/gastric flu. . . .	☐	☐	☐	☐	☐	________	☐	☐	☐	☐	☐	☐☐☐

Continued...

	In which week of pregnancy did you have problems? 13-16	17-20	21-24	25-28	29+	The name of the medication taken	In which week of pregnancy did you take medication 13-16	17-20	21-24	25-28	29+	No. of days taken
11 Unusual fatigue /drowsiness												
12 Heartburn												
13 Swelling of the body (oedema)												
14 Common cold												
15 Throat infection												
16 Sinusitis/ear infection												
17 Influenza												
18 Pneumonia /bronchitis												
19 Other cough												
20 Sugar in urine												
21 Protein in urine												
22 Bladder infection/ cystitis												
23 Incontinence												
24 High blood pressure												
25 Leg cramps												
26 Asthma												
27 Hay fever/other allergy												
28 Headache/migraine												
29 Depression												
30 Other psychological problems												
31 Other												

53. If you have had a fever once or more since the 13th week of pregnancy, indicate in which week of pregnancy, name of any medication taken to reduce the fever and the highest temperature measured. (If more than 3 times, indicate the last 3.)

	Which week of pregnancy did you have a fever? 13–16	17–20	21–24	25–28	29+	Name any medication taken to lower the fever	Highest recorded temperature (*e.g. 38.9° C*)	Temperature not taken
1st time							, °C	
2nd time							, °C	
3rd time							, °C	

☐ Fever more than 3 times

54. Have you taken other medication after the 13th week of pregnancy not previously mentioned, for example, sleeping tablets or sedatives? Give the name, when and how many days altogether the medication was taken **for.** (This applies to all types of medicines including alternative and herbal remedies, both regular and occasional use. Do not include vitamins and nutritional supplements as these are discussed elsewhere.)

**Name of medication** (e.g. Valium, Rohypnol, Paracetamol)	Use of medication in week of pregnancy 13–16	17–20	21–24	25–28	29+	No. of days taken
______	☐	☐	☐	☐	☐	
______	☐	☐	☐	☐	☐	
______	☐	☐	☐	☐	☐	
______	☐	☐	☐	☐	☐	

55. During this pregnancy have you been involved in an accident or been injured *(e.g. traffic accident, fall, hit in the stomach)?*

☐ No

☐ Yes

56. If yes, in which week of pregnancy?

Vitamins, minerals and dietary supplements

57. Have you taken vitamins, minerals or other nutritional supplements after the 13th week of pregnancy?

☐ No (go to question 61)

☐ Yes

If you take supplements, please find the package/bottle.

58. Fill in the table below for the vitamins and minerals found on the vitamin package/bottle. Fill in when and approximately how often you have taken them.

	Week of pregnancy supplement taken? 13–16	17–20	21–24	25–28	29+	How often did you take this supplement? Daily	4-6 times a week	1-3 times a week
1 Folate/folic acid	☐	☐	☐	☐	☐	☐	☐	☐
2 Vitamin B1 (Thiamine)	☐	☐	☐	☐	☐	☐	☐	☐
3 Vitamin B2 (Riboflavin)	☐	☐	☐	☐	☐	☐	☐	☐
4 Vitamin B6 (Pyridoxine)	☐	☐	☐	☐	☐	☐	☐	☐
5 Vitamin B12	☐	☐	☐	☐	☐	☐	☐	☐
6 Niacin	☐	☐	☐	☐	☐	☐	☐	☐
7 Pantothenic acid	☐	☐	☐	☐	☐	☐	☐	☐
8 Biotin	☐	☐	☐	☐	☐	☐	☐	☐
9 Vitamin C	☐	☐	☐	☐	☐	☐	☐	☐
10 Vitamin A	☐	☐	☐	☐	☐	☐	☐	☐
11 Vitamin D	☐	☐	☐	☐	☐	☐	☐	☐
12 Vitamin E	☐	☐	☐	☐	☐	☐	☐	☐
13 Iron	☐	☐	☐	☐	☐	☐	☐	☐
14 Calcium	☐	☐	☐	☐	☐	☐	☐	☐
15 Iodine	☐	☐	☐	☐	☐	☐	☐	☐
16 Zinc	☐	☐	☐	☐	☐	☐	☐	☐
17 Selenium	☐	☐	☐	☐	☐	☐	☐	☐
18 Copper	☐	☐	☐	☐	☐	☐	☐	☐
19 Chromium	☐	☐	☐	☐	☐	☐	☐	☐
20 Magnesium	☐	☐	☐	☐	☐	☐	☐	☐
21 Cod liver oil	☐	☐	☐	☐	☐	☐	☐	☐
22 Omega-3 fatty acid	☐	☐	☐	☐	☐	☐	☐	☐

59. Give the complete name(s) of all the vitamins and nutritional supplements you take. Include also herbal remedies and diet products. (Write clearly using CAPITAL LETTERS since this will be read by a computer.)

1 **e.g.** *V I T A P L E X M E D J E R N*
2
3
4
5
6

60. If you take multivitamins (with or without minerals), do these contain folate/folic acid?

- ☐ No
- ☐ Yes
- ☐ Don't know

WORK

61. Have you been in paid employment during this pregnancy?

- ☐ No *(go to question 76)*
- ☐ Yes

62. Do you have the same job conditions now after the 13th week of pregnancy that you described in the first questionnaire?

- ☐ No
- ☐ Yes *(go to question 66)*

63. If no, in which week of your pregnancy did your work situation change?

Week of pregnancy ☐☐

64. How has your work situation changed?

- ☐ I have stopped working
- ☐ I have gone over to a part-time position
- ☐ Other

65. If you have stopped working, why did you stop?

- ☐ I handed in my notice
- ☐ The work was temporary (seasonal, short-term contract)
- ☐ I was fired
- ☐ Other

66. Have your working arrangements been changed during this pregnancy making your job more suitable for you now that you are pregnant?

- ☐ No
- ☐ Yes

67. If no, why have your working conditions not been changed to make them more suitable for you?

- ☐ Not necessary
- ☐ Impossible or nearly impossible
- ☐ I have asked for changes but no changes have been made
- ☐ It is difficult to ask
- ☐ None of the above (explain why)

68. What are your working hours? (Fill in one or more boxes.)

- ☐ Permanent day work
- ☐ Permanent afternoon or evening work
- ☐ Permanent night work
- ☐ Shift work or shift rotas
- ☐ No set times (extra work, extra shifts, temporary employment, etc.)
- ☐ Other

69. Answer each of the following questions for your present work. (Fill in each item.)

	Yes daily, more than half of working hours	Yes daily, less than half of working hours	Yes periodically, but not daily	Seldom or never
Do you ever have so much to do that your work situation becomes stressful and annoying?	☐	☐	☐	☐
Do you have to bend or turn many times a day?	☐	☐	☐	☐
Do you work with your hands at shoulder level or higher?	☐	☐	☐	☐
Do you work standing or walking about?	☐	☐	☐	☐
In some jobs it is possible to decide yourself how much and how quickly you work. You can, for example, work a little faster one day and take it a little easier the next. Do you have this opportunity?	☐	☐	☐	☐
Is there so much noise at your workplace that it is uncomfortable?	☐	☐	☐	☐
Is there so much noise that you have to raise your voice to speak with others even at a distance of one metre?	☐	☐	☐	☐

70. How often have you worked with a radio transmitter or radar after the 13th week of pregnancy?

- ☐ Seldom/never
- ☐ A few times a week
- ☐ Daily
- ☐ On average, more than 1 hour a day

71. How often have you worked with X-ray equipment (at a distance of less than 2 metres) after the 13th week of pregnancy? *(Do not include treatment as a patient.)*

- ☐ Seldom/never
- ☐ A few times a week
- ☐ Daily
- ☐ On average, more than 1 hour a day

72. Have you been absent from your normal job for more than two weeks after the 13th week of pregnancy?

- ☐ No
- ☐ Yes, part time
- ☐ Yes

73. Are you absent from regular work at the present time?

- ☐ No
- ☐ Yes, part time
- ☐ Yes

74. If yes, why are you currently absent from work?

(Fill in for only one item.)

- ☐ Sick leave (with sick compensation pay)
- ☐ Absent due to sick child
- ☐ Made redundant with compensation
- ☐ Absent with maternity allowance due to the working environment
- ☐ Started maternity leave (with allowance)
- ☐ Service leave
- ☐ Other (describe)

____

____

____

75. Complete the table below if you were on sick leave (full or part time) after the 13th week of pregnancy. Fill in the reason (e.g. pelvic girdle pain, pneumonia), which weeks you were on sick leave, the number of days and the percentage of time each period of sick leave represents. *(Give one reason for sick leave per line.)*

Reason for sick leave	Sick leave during week of pregnancy: 13-16	17-20	21-24	25-28	29+	Number of days	% sick leave
Example: Pelvic girdle pain	☐	☐	☐	☐	☐	*14*	*50*
	☐	☐	☐	☐	☐		
	☐	☐	☐	☐	☐		
	☐	☐	☐	☐	☐		
	☐	☐	☐	☐	☐		

76. Do you currently lift anything over 10 kilos while you are pregnant? *(10 kilos is equivalent to a full bucket of water)*

	Home	Work
Seldom or never	☐	☐
Yes, less than 20 times a week	☐	☐
Yes, more than 20 times a week	☐	☐
Yes, 10-20 times a day	☐	☐
Yes, more than 20 times a day	☐	☐

77. Have others helped you with housework or childcare more than they usually do to relieve you during this pregnancy?

- ☐ Yes, considerably
- ☐ Yes, to a fair extent
- ☐ No, no one has offered
- ☐ No, it has not been necessary

78. If you are on maternity leave for this pregnancy, when did it start?

Date: ☐☐ (day) ☐☐ (month) ☐☐☐☐ (year)

Habits

79. How often do you talk on a mobile phone?

- ☐ Seldom/never
- ☐ A few times a week
- ☐ Daily
- ☐ On average, more than 1 hour a day

80. Do you talk on your mobile phone for longer than 15 minutes at a time?

- ☐ Never
- ☐ Seldom
- ☐ Frequently

81. How frequently have you worked with a computer monitor, laser printer or photocopy machine (at a distance of less than 2 metres) after the 13th week of pregnancy?

	Computer monitor	Laser printer	Photocopy machine
Seldom/never	☐	☐	☐
A few times a week . . .	☐	☐	☐
Daily.	☐	☐	☐
On average, more than 1 hour a day	☐	☐	☐

82. Do you live close to high-voltage power lines?

- ☐ No
- ☐ Yes, closer than 50 metres
- ☐ Yes, between 50 - 100 metres
- ☐ Yes, more than 100 metres

83. How often have you been to a discotheque since you answered the previous questionnaire?

- ☐ Never
- ☐ At least 1-2 times a week
- ☐ Less often

84. How often do you exercise at present? *(Fill in for each item.)*

	Never	1-3 times a month	Once a week	Twice a week	3 times or more a week
1. Walking	☐	☐	☐	☐	☐
2. Brisk walking	☐	☐	☐	☐	☐
3. Running/jogging/orienteering	☐	☐	☐	☐	☐
4. Cycling	☐	☐	☐	☐	☐
5. Training studio/weight training	☐	☐	☐	☐	☐
6. Special gymnastics/aerobics for pregnant women	☐	☐	☐	☐	☐
7. Aerobics/gymnastics/dance without running and jumping	☐	☐	☐	☐	☐
8. Aerobics/gymnastics/dance with running and jumping	☐	☐	☐	☐	☐
9. Dancing (swing/rock/folk)	☐	☐	☐	☐	☐
10. Skiing	☐	☐	☐	☐	☐
11. Ball sports	☐	☐	☐	☐	☐
12. Swimming	☐	☐	☐	☐	☐
13. Riding	☐	☐	☐	☐	☐
14. Other	☐	☐	☐	☐	☐

85. How often do you do exercises at home or at a gym for the following groups of muscles? *(Fill in for each item.)*

	Never	1-3 times a month	Once a week	Twice a week	3 times a week or more
Abdominal muscles	☐	☐	☐	☐	☐
Back muscles	☐	☐	☐	☐	☐
Pelvic floor muscles *(Muscles around the vagina, urethra, anus)*	☐	☐	☐	☐	☐

86. How often at the moment are you so physically active in your spare time and/or at work that you get out of breath or sweat? *(Fill in for both spare time and work.)*

	Spare time	At work
Never	☐	☐
Less than once a week	☐	☐
Once a week	☐	☐
Twice a week	☐	☐
3-4 times a week	☐	☐
5 or more times a week	☐	☐

87. How often on average have you had sexual intercourse during the last month?

- ☐ Daily
- ☐ 5-6 times a week
- ☐ 3-4 times a week
- ☐ 1-2 times a week
- ☐ Less frequently
- ☐ Never

88. Have you been abroad during the last year?

- ☐ No
- ☐ Yes

89. If yes, which countries did you visit and when?

Country	Month	Year
______________	☐☐	☐☐☐☐
______________	☐☐	☐☐☐☐
______________	☐☐	☐☐☐☐

90. Have you come into contact with animals either at work or in your free time?

- ☐ No
- ☐ Yes

91. If yes, which animals have you come into contact with and how often?

	Daily	3–6 times a week	1-2 times a week	Less often
Dog	☐	☐	☐	☐
Cat	☐	☐	☐	☐
Guinea pig/hamster/rabbit/rat, etc.	☐	☐	☐	☐
Canary or other caged birds	☐	☐	☐	☐
Hens and other poultry	☐	☐	☐	☐
Cow/sheep/goat	☐	☐	☐	☐
Horse	☐	☐	☐	☐
Pig	☐	☐	☐	☐
Other	☐	☐	☐	☐

92. How many hours a day do you usually sleep now when you are pregnant?

- ☐ Over 10 hours
- ☐ 8–9 hours
- ☐ 6-7 hours
- ☐ 4-5 hours
- ☐ Less than 4 hours

93. Do you currently sleep on a waterbed or use an electric blanket?

	Yes	No
Waterbed	☐	☐
Electric blanket	☐	☐

94. Can you rest during the day (both at home and at work)?

- ☐ No
- ☐ Yes

95. Have you been in a sauna while you have been pregnant?

- ☐ No
- ☐ 1–5 times
- ☐ 6-10 times
- ☐ More than 10 times

96. Have you been in a solarium while you have been pregnant?

- ☐ No
- ☐ 1-5 times
- ☐ 6-10 times
- ☐ More than 10 times

97. Are you exposed to passive smoking either at home or at work? If yes, how many hours a day?

	No	Yes	No. of hrs
Home	☐	☐	☐☐
Work	☐	☐	☐☐

98. Do you smoke at present? If yes, how many cigarettes?

- ☐ No
- ☐ Sometimes ☐☐ Cigarettes per week
- ☐ Daily ☐☐ Cigarettes per day

99. Does the baby's father smoke at present? If yes, how many cigarettes?

- ☐ No
- ☐ Sometimes ☐☐ Cigarettes per week
- ☐ Daily ☐☐ Cigarettes per day

100. If one or both of you have stopped smoking during the pregnancy, in which week of pregnancy did you stop?

- ☐ You ☐☐ Week of pregnancy
- ☐ Baby's father ☐☐ Week of pregnancy

101. If you or the baby's father have smoked during the pregnancy, were there periods during which you or the baby's father did not smoke? *(Fill in the weeks during pregnancy when you did not smoke.)*

Weeks of pregnancy without smoking

	0–4	5–8	9–12	13–16	17–20	21–24	25–28	29+
You	☐	☐	☐	☐	☐	☐	☐	☐
Baby's father	☐	☐	☐	☐	☐	☐	☐	☐

102. Have you used other forms of nicotine after the 13th week of pregnancy?

	No	Yes
Nicotine chewing gum	☐	☐
Nicotine patches	☐	☐
Nicotine inhaler	☐	☐
Chewing tobacco/snuff	☐	☐

103. Have you used any of the following substances after the 13th week of pregnancy?

	No	Yes
Hash	☐	☐
Amphetamine	☐	☐
Ecstasy	☐	☐
Cocaine	☐	☐
Heroin	☐	☐

104. Have you ever used any of the following substances? (Fill in for each item.)

	No	Previously	Last 6 months before pregnancy	During this pregnancy
Anabolic steroids	☐	☐	☐	☐
Testosterone products	☐	☐	☐	☐
Growth hormones *(e.g. genotropin/somatropin)*	☐	☐	☐	☐

Food and drink

105. How often do you eat the following foods? *(Fill in for each item.)*

		Before the pregnancy				During the pregnancy		
		Never	A few times a year	1–3 times a Month	Once a week or more	Never	1–3 times a month	Once a week or more
1	Crab	☐	☐	☐	☐	☐	☐	☐
2	Shrimps	☐	☐	☐	☐	☐	☐	☐
3	Shellfish (e.g. mussels, oysters)	☐	☐	☐	☐	☐	☐	☐
4	Fish liver	☐	☐	☐	☐	☐	☐	☐
5	Tuna fish or halibut	☐	☐	☐	☐	☐	☐	☐
6	Flounder/other flat fish	☐	☐	☐	☐	☐	☐	☐
7	Pike or perch	☐	☐	☐	☐	☐	☐	☐
8	Other fresh water fish	☐	☐	☐	☐	☐	☐	☐
9	Reindeer meat	☐	☐	☐	☐	☐	☐	☐
10	Mutton	☐	☐	☐	☐	☐	☐	☐
11	Liver or kidney from game	☐	☐	☐	☐	☐	☐	☐
12	Wild mushrooms	☐	☐	☐	☐	☐	☐	☐

106. How often do you eat the following types of food? *(Fill in for each item.)*

	Never	A few times a year	1-3 times a month	Once a week or more
Food from restaurant/street vendors/canteen or the like	☐	☐	☐	☐
Meat (not including tinned) bought in other countries	☐	☐	☐	☐
Meat (including poultry) that is raw or undercooked (pink near the bone)	☐	☐	☐	☐
Raw minced meat/meat mixtures (even to taste)	☐	☐	☐	☐
Smoked or cured salmon or trout (uncooked)	☐	☐	☐	☐
Soft cheeses (e.g. cream cheese, camembert, blue cheese, etc.)	☐	☐	☐	☐
Unwashed raw vegetables, unwashed fruit	☐	☐	☐	☐

107. Do you avoid eating the following foods during this pregnancy?

	No	Yes
Fish	☐	☐
Eggs	☐	☐
Nuts	☐	☐
Oranges, lemons	☐	☐
Strawberries	☐	☐

Other, specify ______________________

108. What type of drinking water do you have where you live?

☐ Own water source (e.g. well)
☐ Water company (public or private)
☐ Other source

Name of water company ______________________

☐ Don't know the name of the water company

109. Is your water treated (chlorinated or UV-radiated)?

☐ No
☐ Yes, UV radiation
☐ Yes, chlorinated
☐ Don't know

110. What was your fluid consumption (number of cups/glasses) per day after the 13th week of pregnancy? *(1 mug = 2 cups, 1 small plastic bottle (0.5 litre) = 4 cups, 1 large plastic bottle (1.5 litres) = 12 cups)*

	Number of cups / glasses	Decaffeinated (fill in)
1. Filter coffee	☐☐	☐
2. Instant coffee	☐☐	☐
3. Boiled coffee	☐☐	☐
4. Other coffee	☐☐	☐
5. Tea	☐☐	☐
6. Coca Cola/Pepsi, etc.	☐☐	☐
7. Other fizzy drinks	☐☐	☐
8. Diet Coca Cola, diet Pepsi	☐☐	☐
9. Other diet fizzy drinks	☐☐	☐
10. Tap water	☐☐	
11. Bottled water	☐☐	

	Number of cups/glass	Organic (fill in)
12. Juice/squash		☐
13. Diet juice/squash		☐
14. Milk (skimmed, low fat, whole)		☐
15. Yogurt, all types.		☐
16. Yogurt with active Lactobacillus all types		☐
17. Other type of cultured milk (kefir)		☐
18. Other.		☐

111. How often did you consume alcohol before and how often do you consume it now?

	Last 3 months before last period	In this pregnancy week of pregnancy 0–12	13–24	25+
Roughly 6-7 times a week .	☐	☐	☐	☐
Roughly 4–5 times a week	☐	☐	☐	☐
Roughly 2-3 times a week .	☐	☐	☐	☐
Roughly 1 time a week	☐	☐	☐	☐
Roughly 1–3 times a month	☐	☐	☐	☐
Less then once a month . . .	☐	☐	☐	☐
Never.	☐	☐	☐	☐

Alcohol units
Alcohol units are used to compare the different types of alcoholic beverages. 1 alcohol unit = 1.5 cl. pure alcohol.

1 glass of beer	= 1 alcohol unit
1 wine glass of red or white wine	= 1 alcohol unit
1 sherry glass of sherry or other fortified wine	= 1 alcohol unit
1 spirit glass of spirits or liqueur	= 1 alcohol unit
1 bottle/can breezer or cider	= 1 alcohol unit

112. In the period just before you became pregnant and during this pregnancy, how many times have you consumed 5 units or more of alcohol? *(See the explanation for units.)*

	Last 3 mths before last period	In this pregnancy week of pregnancy 0–12	13–24	25+
Several times a week	☐	☐	☐	☐
Once a week	☐	☐	☐	☐
1-3 times a month	☐	☐	☐	☐
Less than once a month . . .	☐	☐	☐	☐
Never	☐	☐	☐	☐

113. How many units do you usually drink when you consume alcohol? *(See the above explanation.)*

	Last 3 mths before last period	In this pregnancy week of pregnancy 0–12	13–24	25+
10 or more	☐	☐	☐	☐
7–9	☐	☐	☐	☐
5–6	☐	☐	☐	☐
3–4	☐	☐	☐	☐
1–2	☐	☐	☐	☐
Less than 1	☐	☐	☐	☐

114. If you have changed your drinking habits before this pregnancy, when did the change occur? *(Fill in one or more boxes.)*

	Reduced intake	Increased intake
Last 3 months before last period	☐	☐
During pregnancy weeks 0-6	☐	☐
During pregnancy weeks 7-12	☐	☐
During pregnancy weeks 13-24	☐	☐
After pregnancy week 25	☐	☐

115. If you have modified your consumption of alcohol, how important were the following factors? *(Fill in one or more boxes.)*

	Not relevant	Not very important	Quite important	Important	Very important
Nausea, discomfort . .	☐	☐	☐	☐	☐
Altered taste	☐	☐	☐	☐	☐
For the baby's sake . .	☐	☐	☐	☐	☐
Depression/problems .	☐	☐	☐	☐	☐
Other reasons	☐	☐	☐	☐	☐

You and your life now

116. What is your current civil status?

☐ Married
☐ Cohabiting
☐ Single
☐ Divorced/separated
☐ Widowed
☐ Other

117. Do you have anyone other than your husband/partner you can ask for advice in a difficult situation?

☐ No.
☐ Yes, 1 or 2 people
☐ Yes, more than 2 people

118. How frequently do you meet or talk on the telephone with your family (other than your husband/partner and children) or close friends?

☐ Once a month or less
☐ 2-8 times a month
☐ More than twice a week

119. Do you often feel lonely?

☐ Almost never
☐ Seldom
☐ Sometimes
☐ Usually
☐ Almost always

120. If you have given birth before, in general, how was the experience of giving birth?

☐ Very good
☐ Good
☐ Alright
☐ Bad
☐ Very bad

121. Do you agree or disagree with the following statements relating to the forthcoming birth of your baby?
(Fill in for each statement.)

	Agree completely	Agree	Agree somewhat	Disagree somewhat	Disagree	Disagree completely
I want to give birth as naturally as possible without painkillers or intervention	☐	☐	☐	☐	☐	☐
I am really dreading giving birth	☐	☐	☐	☐	☐	☐
I want to have enough medication so that the birth will be painless	☐	☐	☐	☐	☐	☐
I want to have an epidural regardless	☐	☐	☐	☐	☐	☐
I want to have an epidural if the midwife agrees	☐	☐	☐	☐	☐	☐
If I could choose I would have a caesarean	☐	☐	☐	☐	☐	☐
I think the woman herself should decide whether or not to have a caesarean	☐	☐	☐	☐	☐	☐
I worry all the time that the baby will not be healthy or normal	☐	☐	☐	☐	☐	☐
I am really looking forward to the baby coming	☐	☐	☐	☐	☐	☐

122. How do these statements describe your relationship? (Only answer if you have a partner.)
(Fill in for each statement.)

	Agree completely	Agree	Agree somewhat	Disagree somewhat	Disagree	Completely disagree
My husband/partner and I have a close relationship	☐	☐	☐	☐	☐	☐
My partner and I have problems in our relationship	☐	☐	☐	☐	☐	☐
I am very happy in my relationship	☐	☐	☐	☐	☐	☐
My partner is usually understanding	☐	☐	☐	☐	☐	☐
I often think about ending our relationship	☐	☐	☐	☐	☐	☐
I am satisfied with my relationship with my partner	☐	☐	☐	☐	☐	☐
We often disagree about important decisions	☐	☐	☐	☐	☐	☐
I have been lucky in my choice of a partner	☐	☐	☐	☐	☐	☐
We agree on how children should be raised	☐	☐	☐	☐	☐	☐
I think my partner is satisfied with our relationship	☐	☐	☐	☐	☐	☐

123. Have you been bothered during the last 2 weeks by any of the following? *(Enter a cross in a box for each item.)*

	Not bothered	Slightly bothered	Fairly much bothered	Very much bothered
1. Feeling fearful	☐	☐	☐	☐
2. Nervousness or shakiness inside	☐	☐	☐	☐
3. Feeling hopeless about the future	☐	☐	☐	☐
4. Feeling blue	☐	☐	☐	☐
5. Worrying too much about things	☐	☐	☐	☐
6. Feeling everything is an effort	☐	☐	☐	☐
7. Feeling tense or keyed up	☐	☐	☐	☐
8. Suddenly scared for no reason	☐	☐	☐	☐

124. How often do you experience the following in your everyday life? *(Fill in for each statement.)*

	Seldom/ never	Fairly seldom	Sometimes	Often	Very often
Feel pleased about something	☐	☐	☐	☐	☐
Feel happy	☐	☐	☐	☐	☐
Feel joyful, as though everything is going your way	☐	☐	☐	☐	☐
Feel that you will scream at someone or hit something	☐	☐	☐	☐	☐
Feel angry, irritated or annoyed	☐	☐	☐	☐	☐
Feel mad at someone	☐	☐	☐	☐	☐

125. How well do these statements describe you? *(Fill in for each statement.)*

	Incorrect	Partly correct	Almost correct	Completely correct
I always manage to solve difficult problems if I try hard enough	☐	☐	☐	☐
If anyone opposes me, I find a way to get what I want	☐	☐	☐	☐
I am sure that I can cope with unexpected events	☐	☐	☐	☐
I am calm when I encounter difficulties because I trust my ability to cope	☐	☐	☐	☐
When I am in a difficult situation, I usually find a solution	☐	☐	☐	☐

126. Do you agree or disagree with the following statements? *(Fill in for each statement.)*

	Disagree completely	Disagree	Disagree somewhat	Don't agree or disagree	Agree somewhat	Agree	Agree completely
My life is largely what I wanted it to be	☐	☐	☐	☐	☐	☐	☐
My life is very good	☐	☐	☐	☐	☐	☐	☐
I am satisfied with my life	☐	☐	☐	☐	☐	☐	☐
I have achieved so far what is important for me in my life	☐	☐	☐	☐	☐	☐	☐
If I could start all over, there is very little I would do differently	☐	☐	☐	☐	☐	☐	☐

127. How do you feel about yourself? *(Fill in for each statement.)*

	Agree completely	Agree	Disagree	Disagree completely
I have a positive attitude toward myself	☐	☐	☐	☐
I feel completely useless at times	☐	☐	☐	☐
I feel that I do not have much to be proud about	☐	☐	☐	☐
I feel that I am a valuable person, as good as anyone else	☐	☐	☐	☐

128. Have you experienced any of the following during the last 12 months? If yes, how painful or difficult was it for you?
(Fill in for each statement.)

			IF YES		
	No	Yes	Not too bad	Painful/ difficult	Very painful/ difficult
Have you had problems at work or where you study?	☐	☐	☐	☐	☐
Have you had financial problems?	☐	☐	☐	☐	☐
Have you been divorced, separated or ended your relationship with your partner?	☐	☐	☐	☐	☐
Have you had problems or conflicts with your family, friends or neighbours?	☐	☐	☐	☐	☐
Have you been seriously ill or injured?	☐	☐	☐	☐	☐
Has anyone close to you been seriously ill or injured?	☐	☐	☐	☐	☐
Have you been involved in a serious accident, fire or robbery?	☐	☐	☐	☐	☐
Have you lost someone close to you?	☐	☐	☐	☐	☐
Other	☐	☐	☐	☐	☐

129. Have you ever experienced any of the following? *(Fill in for each statement.)*

				Who was responsible for this?			Has this occurred during the last year?	
	No, never	Yes, as a child *(under 18)*	Yes, as an adult *(over 18)*	A stranger	Family or relative	Another known person	No	Yes
Someone has over a long period of time systematically tried to subdue, degrade or humiliate you	☐	☐	☐	☐	☐	☐	☐	☐
Someone has threatened to hurt you or someone close to you	☐	☐	☐	☐	☐	☐	☐	☐
You have been subjected to physical abuse	☐	☐	☐	☐	☐	☐	☐	☐
You have been forced to have sexual intercourse	☐	☐	☐	☐	☐	☐	☐	☐

Miscellaneous

130. Has anyone living with you had any of the following illnesses during this pregnancy? *(Enter a cross and specify the period)*

	In which week of pregnancy? 0–9	10–19	20–29	30+
☐ Influenza	☐	☐	☐	☐
☐ Childhood diseases (fever and rash)	☐	☐	☐	☐
☐ Prolonged cough	☐	☐	☐	☐
☐ Other infectious disease	☐	☐	☐	☐

131. Have there been any instances of cot death in your family or your partner's family?

☐ No
☐ Don't know
☐ Yes, in my family (see question 132)
☐ Yes, in the baby's father's family (see question 133)

132. The child that died of cot death in my family was:

☐ My sister
☐ My brother
☐ My sister's child ☐ Boy ☐ Girl
☐ My brother's child ☐ Boy ☐ Girl
☐ My mother's sibling ☐ Boy ☐ Girl ☐ Sex unknown
☐ My father's sibling ☐ Boy ☐ Girl ☐ Sex unknown
☐ Other

133. The child that died of cot death in the baby's father's family was:

☐ Baby's father's sister
☐ Baby's father's brother
☐ Baby's father's sister's child ☐ Boy ☐ Girl
☐ Baby's father's brother's child ☐ Boy ☐ Girl
☐ Baby's paternal grandmother's sibling ☐ Boy ☐ Girl ☐ Sex unknown
☐ Baby's paternal grandfather's sibling ☐ Boy ☐ Girl ☐ Sex unknown
☐ Other

134. Have you ever lost a child?

☐ No *(if no, you are finished with the questionnaire)*
☐ Yes

135. If yes, what was the cause of death and when did the death occur?

☐ Stillbirth (Birth after the 16th week of pregnancy.)
☐ Cot death
☐ Accident
☐ Illness/birth defect
Which illness/birth defect: ____________________

☐ Other

	Year	Child's age: Years	Child's age: Months
Child 1			
Child 2			

136. Did you receive counselling from healthcare staff or other persons after the death? How many sessions did you have with healthcare staff, and/or parent support group, family and friends? How many weeks did you receive support?

	Healthcare staff	Parent support group, family, friends
Number of meetings (approximately):		
Number of sessions via telephone (approximately):		
Weeks of support (approximately):		

137. Do you feel that the follow -up you received after your child's death was adequate?

☐ No follow-up was provided
☐ Very good
☐ Good enough
☐ Should have been better
☐ Bad

138. Has the death made you more anxious during this pregnancy?

☐ No, not at all
☐ No, not very much
☐ Yes, to a fair extent
☐ Yes, very much

139. Do you feel that the health care staff at the antenatal clinics took into consideration this painful experience in their contact with you?

☐ Yes, very much
☐ Yes, to a fair extent
☐ No, not at all

Have you remembered to fill in the date on which you completed the questionnaire on page 1?

Thank you very much for your help!

Please return the completed questionnaire in the stamped addressed envelope provided.
