# Supplementary material for: Genetic testing of newborns for type 1 diabetes susceptibility: a prospective cohort study on effects on maternal mental health
Source: BMC Med Genet. 2010 Jul 15;11:112. doi: 10.1186/1471-2350-11-112 (PMC3152763; doi:10.1186/1471-2350-11-112)
Supplement: Additional file 2 — 6 month questionnaire. The MoBa questionnaire when the child is 6 months old. SWLS corresponds to item no. 90, "Maternal worry about the child" to item 91, RSES to item 93, and SCL-8 to item 94. [file 1471-2350-11-112-S2.PDF]

den norske **Mor & barn undersøkelsen**

Questionnaire 4 - When your child is around 6 months old

This questionnaire comes in two parts. The first part is about your child, while the other part is about yourself. It will help if you have your child's health card to hand before you start answering the questions so that you can use the information contained in it when completing this questionnaire. If you find a question difficult to answer, you can skip it and go onto the next question.

If you have had twins or triplets, complete one questionnaire for each child.

The questionnaire will be processed by a computer. ***It is therefore important that you follow these instructions when completing it:***

- Use a blue or black ballpoint pen.
- In the small check boxes, enter *a cross* to indicate what you think is the most appropriate answer like this: ☒
  If you make a mistake you can delete the cross by filling in the box completely like this: ■
- Write numbers in the large green boxes.

  It is important that you only write in the white area of each box like this:

  Number: 0 1 2 3 4 5 6 7 8 9
- In the case of numbered boxes with more than one square, enter a one-digit number in the right box. *Example: 5 is entered as follows* [ ][5]
- Date boxes are split into 3 sections, with the first one for the day of the month, the second one for the month and the last one for the year.

  So, enter the date as follows: [6] Day [5] Month [2005] Year
- Specific information concerning, *for example, medication* should be written on the lines provided. *Please write clearly!*

As soon as you have completed the questionnaire, return it to us in the enclosed stamped addressed envelope.

Specify the day, month and year when the questionnaire was completed [ ][ ] Day [ ][ ] Month [ ][ ][ ][ ] Year *(write the year in full, e.g. 2005)*

About your child's birth

1. Is your child a boy or girl?

☐ Boy

☐ Girl

2. How big was your child when he/she was born?

Birth weight: [ ][ ][ ][ ] g

Length: [ ][ ],[ ] cm

3. In which week of your pregnancy did you give birth?

[ ][ ] week

4. How long was your child in hospital after the birth?

Number of days [ ][ ] or weeks [ ][ ]

5. Was your child transferred to another department or hospital after the birth?

☐ No

☐ Yes

If yes, specify ________________________________

6. Was your child delivered by caesarean section?

☐ No

☐ Yes

Sp.skj. 4 Engelsk 4G MB 1.000 05.08 - Bording

7. If yes, was the caesarean section planned?

- ☐ No
- ☐ Yes

If yes, why?

- ☐ Breech presentation
- ☐ Previous caesarean
- ☐ Pregnancy complication or mother taken ill
- ☐ Poor growth or other factor relating to the foetus
- ☐ Own preference
- ☐ Other

8. Were there any complications during the birth?

- ☐ No
- ☐ Yes

If so, describe:______________________________

9. Were you admitted or transferred to another department or other hospital due to complications in connection with the birth? *(Applies both before and after the birth.)*

- ☐ No
- ☐ Yes

10. If yes, where?

Department:

____

Hospital: ______________________________

11. How many days were you in hospital in connection with the birth?

Before the birth ☐☐ Number of days

After the birth ☐☐ Number of days

12. Did the birth go as you had expected?

- ☐ Yes, as expected
- ☐ No, it went better
- ☐ Neither/nor
- ☐ No, it was worse
- ☐ Don't know

13. How true do you think the following descriptions are of the birth? *(Enter a cross in a box for each item.)*

	Fairly true	Partially true	Not true
I felt safe and in good hands	☐	☐	☐
I was a lot of pain	☐	☐	☐
I received too few pain-killing drugs	☐	☐	☐

14. Was anyone from your close family present at the birth?

- ☐ Yes, child's father
- ☐ Yes, someone else
- ☐ No

About your child

Nutrition

15. What did you give your child to drink <u>during the first week of life</u>?
(You can enter a cross in more than one box.)

- ☐ Breast milk
- ☐ Water
- ☐ Sugar water
- ☐ Formula
- ☐ Other, specify: ______________________________
- ☐ Don't know/don't remember

16. What has your child been given to drink during the first <u>6 months of his/her life</u>?
(Enter a cross for each month you gave your child the relevant drink.)

	Child's age in months 0	1	2	3	4	5	6
Breast milk	☐	☐	☐	☐	☐	☐	☐
Standard Collett formula .	☐	☐	☐	☐	☐	☐	☐
Collett formula with Omega 3	☐	☐	☐	☐	☐	☐	☐
Standard NAN formula ..	☐	☐	☐	☐	☐	☐	☐
Nan HA1 formula	☐	☐	☐	☐	☐	☐	☐
Other milk, specify: ____________________	☐	☐	☐	☐	☐	☐	☐
Water	☐	☐	☐	☐	☐	☐	☐
Squash/Juice	☐	☐	☐	☐	☐	☐	☐

17. How often do you give your child the following to drink at the moment? *(Enter a cross in a box for each item.)*

	Never/ seldom	1-3 times a week	4-6 times a week	At least once a day
1. Breast milk	☐	☐	☐	☐
2. Breast milk supplement	☐	☐	☐	☐
3. Normal sweet milk, any type	☐	☐	☐	☐
4. sour milk (yogurt, buttermilk, etc.)	☐	☐	☐	☐
5. Organic milk products (milk, yogurt)	☐	☐	☐	☐
6. Boiled water	☐	☐	☐	☐

Cont.

	Never/ seldom	1-3 times a week	4-6 times a week	At least once a day
7. Tap water	☐	☐	☐	☐
8. Bottled water	☐	☐	☐	☐
9. Bottled baby cordial	☐	☐	☐	☐
10. Other type of cordial, sweetened	☐	☐	☐	☐
11. Cordial, artificially sweetened	☐	☐	☐	☐
12. Juice	☐	☐	☐	☐
13. Other, specify: ________________	☐	☐	☐	☐

18. How often does your child eat the following food at the moment, and how old was your child when you started giving him/her this food?

	How often do you give this to your child?				How old was your child when you gave him/her this foodfor the first time?
	Never/ seldom	1-3 times a week	4-6 times a week	At least once a day	
**Instant porridge**					
1. Rice porridge, maize porridge	☐	☐	☐	☐	☐ months
2. Oatmeal porridge, different types	☐	☐	☐	☐	☐ months
3. Wheat porridge, all types, rusk porridge	☐	☐	☐	☐	☐ months
**Home-made porridge using:**					
4. Wheat flour (rough/fine), rusk, semolina, oats	☐	☐	☐	☐	☐ months
5. Iron-enriched wheat flour	☐	☐	☐	☐	☐ months
6. Helios baby flour	☐	☐	☐	☐	☐ months
7. Millet	☐	☐	☐	☐	☐ months
**Processed dinner in a jar:**					
8. Vegetables	☐	☐	☐	☐	☐ months
9. Vegetables and meat	☐	☐	☐	☐	☐ months
**Home-made dinner:**					
10. Potato/vegetable puree	☐	☐	☐	☐	☐ months
11. Meat and vegetables/potatoes	☐	☐	☐	☐	☐ months
12. Fish and vegetables/potatoes	☐	☐	☐	☐	☐ months
13. Other type of home-made dinner	☐	☐	☐	☐	☐ months
**Snack/dessert:**					
14. Home-made fruit puree	☐	☐	☐	☐	☐ months
15. Fruit/berry puree in a jar	☐	☐	☐	☐	☐ months
16. Rusks/biscuits/bread	☐	☐	☐	☐	☐ months
17. Other, specify: ____________	☐	☐	☐	☐	☐ months

19. Do you think or do you know that your child has a reaction to milk/dairy products?

- ☐ No
- ☐ Yes

20. If yes, which products?

- ☐ Whole milk
- ☐ Low-fat milk/skimmed milk
- ☐ Cream/whipped cream/ice cream
- ☐ Yogurt/sour milk
- ☐ Breast milk when mother is drinking milk
- ☐ Other

21. Do you give your child cod liver oil, vitamins, iron or any other dietary supplement?

☐ No ☐ Yes

22. If you give your child cod liver oil, vitamins, iron or another dietary supplement, specify how much you give your child each time and how often. How old was your child in months and weeks when you gave him/her the product for the first time?

Name of product	How many teaspoons each time?	How often do you give your child this?		How old was your child when you started giving the product?	
1. Cod liver oil	☐ teaspoons	☐ daily	☐ sometimes	☐ months and	☐ weeks
2. Biovit	☐ teaspoons	☐ daily	☐ sometimes	☐ months and	☐ weeks
3. Sanasol	☐ teaspoons	☐ daily	☐ sometimes	☐ months and	☐ weeks
4. Nycoplus Multi-Vitamin mixture for children	☐ teaspoons	☐ daily	☐ sometimes	☐ months and	☐ weeks
5. Fluoride		☐ daily	☐ sometimes	☐ months and	☐ weeks
6. Iron supplement, specify: ____________________		☐ daily	☐ sometimes	☐ months and	☐ weeks
7. Other dietary supplement, specify: ____________________		☐ daily	☐ sometimes	☐ months and	☐ weeks

Growth, health and use of medication

You will find the information to help you answer the following questions on your child's health card.

23. How many times have you been to the mother and child health centre with your child?

- ☐ Never
- ☐ 1-2 times
- ☐ 3-5 times
- ☐ 6-10 times
- ☐ more than 10 times

24. Has your child been given the vaccinations recommended by the health centre?

- ☐ Yes
- ☐ No, don't want vaccination
- ☐ No, your child has been often ill
- ☐ No, vaccinations postponed for practical reasons
- ☐ Don't know

25. Referring to your child's health card, enter a cross for the vaccinations which your child has received and whether the vaccinations had any side-effect. *(Enter a cross in a box for each item.)*

	Has your child received the vaccination?		Was there any side-effect after the vaccination?		Was there any side-effect resulting in contact with a doctor?		Was there any side-effect resulting in hospital admission?	
**Vaccinations**	No	Yes	No	Yes	No	Yes	No	Yes
1. DTP (Infanrix)	☐	☐	☐	☐	☐	☐	☐	☐
2. DT (diphtheria/tetanus)	☐	☐	☐	☐	☐	☐	☐	☐
3. Polio – Hib (Act-Hib polio)	☐	☐	☐	☐	☐	☐	☐	☐
4. Hepatitis B (Engerix-B)	☐	☐	☐	☐	☐	☐	☐	☐
5. BCG (tuberculosis)	☐	☐	☐	☐	☐	☐	☐	☐
6. Pneumococcus (Prevenar)	☐	☐	☐	☐	☐	☐	☐	☐
7. Other vaccination: ____________	☐	☐	☐	☐	☐	☐	☐	☐

26. Referring to your child's health card, enter below your child's weight, length and head circumference when he/she was around 6 weeks, 3 months and 6 months.

	Date of examination					
	Day	Month	Year	Length	Head circumference	Weight
**Approx. 6 weeks**	☐☐	☐☐	☐☐	☐☐,☐ cm	☐☐,☐ cm	☐☐☐☐ g
**Approx. 3 months**	☐☐	☐☐	☐☐	☐☐,☐ cm	☐☐,☐ cm	☐☐☐☐ g
**5-6 months**	☐☐	☐☐	☐☐	☐☐,☐ cm	☐☐,☐ cm	☐☐☐☐☐ g

The following questions concern any illnesses or health problems your child has had. We will first ask you about more longterm problems, then about illnesses and problems of a more acute nature.

27. Does your child have or has he/she had any of the following health problems? If yes, has the mother and child health centre or someone else referred your child for further specialist investigation? *(Enter a cross in a box for each item.)*

	Has(had) your child problems?		Has your child been referred for a specialist investigation?		
	No	Yes	No	Yes, referred from health centre	Yes, referred by someone else
1. Hip disorder/dislocated hip	☐	☐	☐	☐	☐
2. Impaired hearing	☐	☐	☐	☐	☐
3. Impaired vision	☐	☐	☐	☐	☐
4. Delayed motor development (movement development)	☐	☐	☐	☐	☐
5. Too little weight gain	☐	☐	☐	☐	☐
6. Too much weight gain	☐	☐	☐	☐	☐
7. Abnormal head circumference	☐	☐	☐	☐	☐
8. Heart defect	☐	☐	☐	☐	☐
9. Testicles not descended into scrotum	☐	☐	☐	☐	☐
10. Asthma	☐	☐	☐	☐	☐
11. Atopic eczema (childhood eczema)	☐	☐	☐	☐	☐
12. Hives	☐	☐	☐	☐	☐
13. Food allergy/intolerance	☐	☐	☐	☐	☐
14. Delayed psychomotor development (several functions)	☐	☐	☐	☐	☐
15. (Other) malformations: ___________________________	☐	☐	☐	☐	☐
16. Other:____	☐	☐	☐	☐	☐

28. If your child was referred for a specialist investigation, what did this investigation show?

☐ Everything was fine

☐ Still some doubts/further investigations needed

☐ Don't know

☐ Given the following diagnosis: ________________________

____

____

29. Is you child suspected of having a syndrome or chromosomal defect?

☐ No

☐ Yes, a syndrome

☐ Yes, a chromosomal defect

☐ If yes, specify the name or describe the problem:________

____

____

30. Has your child been treated for a hip problem (hip dysplasia)?

☐ No

☐ Yes, treated with a cushion

☐ Yes, treated with a plaster cast

☐ Yes, treated with braces

If yes, how long did the treatment go on for? ☐ months

31. Has your child had the following illness/health problem? If yes, did you go to a doctor or hospital about it? *(Enter a cross in a box for each item.)*

	Has your child had health problems?		Number of times	Did you go to a doctor/clinic for this?		Has your child been admitted to hospital for this?	
	No	Yes		No	Yes	No	Yes
1. Common cold	☐	☐		☐	☐	☐	☐
2. Throat infection	☐	☐		☐	☐	☐	☐
3. Ear infection	☐	☐		☐	☐	☐	☐
4. Pseudocroup	☐	☐		☐	☐	☐	☐
5. Bronchitis/RS virus/pneumonia	☐	☐		☐	☐	☐	☐
6. Gastric flu/diarrhoea	☐	☐		☐	☐	☐	☐
7. Urinary tract infection	☐	☐		☐	☐	☐	☐
8. Conjunctivitis	☐	☐		☐	☐	☐	☐
9. Febrile convulsions	☐	☐		☐	☐	☐	☐
10. Other convulsions (without any fever)	☐	☐		☐	☐	☐	☐
11. Colic	☐	☐		☐	☐	☐	☐
12. Nappy rash	☐	☐		☐	☐	☐	☐
13. Other, describe ______________________	☐	☐		☐	☐	☐	☐

32. Have your child ever been given any medication?

☐ No
☐ Yes

33. If yes, give the name of the medicines and when they were given. *(Include all types of medication, as well as natural medicines, taken both on a regular and occasional basis.)*

**Name of medicine** *(e.g. Apocilin, Paracetamol)*	How old was your child when you gave the medicine? <1 Month	1-2 months	3-4 months	5-6 months	Number of days given in total
______________________	☐	☐	☐	☐	
______________________	☐	☐	☐	☐	
______________________	☐	☐	☐	☐	
______________________	☐	☐	☐	☐	

34. Has your child been examined at or admitted to hospital (since returning home from hospital after birth)?

☐ No

☐ Yes, specify: ____

35. Has your child been operated on or does he/she have a condition requiring an operation?

☐ No

☐ Yes, specify:

____

Development, childcare and life style

36. The following questions concern your child's development. If you haven't actually observed your child, spend a little time looking at what he/she can actually do. *(Enter a cross in a box for each question.)*

	Yes often	Yes, but seldom	No, not yet	Don't know
1. When your child is lying on his/her back, does he/she play by grabbing hold of his/her feet?	☐	☐	☐	☐
2. When your child is lying on his/her tummy, does he/she raise his/her upper body off the ground with straight arms?	☐	☐	☐	☐
3. Does your child roll over from his/her back onto his/her tummy?	☐	☐	☐	☐
4. When you "chat" to your child, does he/she try to "chat" back to you?	☐	☐	☐	☐
5. Does your child babble and make sounds when he/she is lying on his/her own?	☐	☐	☐	☐
6. Can you tell how your child is just by listening to the sounds he/she is making *(e.g. contented, hungry, angry,in pain)?*	☐	☐	☐	☐
7. Do you get a smile from your child when you just smile at him/her *(without touching or tickling him/her and without holding up a toy)?*	☐	☐	☐	☐
8. When you call your child, does he/she turn towards you one of the first times you say his/her name?	☐	☐	☐	☐
9. Does your child grab hold of a toy you give him/her and then put it in his/her mouth or hold it?	☐	☐	☐	☐
10. When your child is sitting on your lap, does he/she stretch out for a toy or something else on the table in front of you?	☐	☐	☐	☐
11. Does your child hold onto a toy with both hands when he/she is examining it?	☐	☐	☐	☐

37. Where is your child cared for during the day?

☐ At home with mother/father/other family member
☐ At home with an unqualified childminder
☐ At a childminder's/family creche
☐ In an outdoor nursery
☐ In a nursery

38. How many other children are there usually along with your child during the day?

☐☐ children

39. Does your child go to baby swimming?

☐ No
☐ Yes

If yes, indicate the number of times during the last 2 months ☐☐

40. How often is your child outside? *(Enter just one cross.)*

☐ Seldom
☐ Often, but less than 1 hour a day
☐ 1-3 hours a day
☐ More than 3 hours a day

41. Does your child use a dummy/pacifier?

☐ Seldom or never
☐ Only when he/she goes to sleep
☐ Often
☐ Most of the time

42. How many hours in total does your child sleep per 24 hours?

☐ Less than 8 hours
☐ 8 - 10 hours
☐ 11 - 13 hours
☐ 13 - 14 hours
☐ More than 14 hours

43. How do you put your child down when he/she is going to sleep? *(Enter a cross in a box for each item.)*

	On back	On side	On tummy
After the birth	☐	☐	☐
At 2 months	☐	☐	☐
At 4 months	☐	☐	☐
At 6 months	☐	☐	☐

44. Does your child share a bed with his/her mother/father (at least half the night)? *(Enter a cross in a box for each item.)*

	No	sometimes	Often
After the birth	☐	☐	☐
At 2 months	☐	☐	☐
At 4 months	☐	☐	☐
At 6 months	☐	☐	☐

45. Enter a cross to indicate whether you agree or disagree with the following statements about your child's mood and temperament. Think about how he/she usually is. *(Enter a cross in a box for each item.)*

	Totally disagree	Disagree	Slightly disagree	Neither agree or disagree	Slightly agree	Agree	Totally agree
1. Your child whimpers and cries a lot	☐	☐	☐	☐	☐	☐	☐
2. Your child is usually easy to pacify when he/she is crying	☐	☐	☐	☐	☐	☐	☐
3. It doesn't take much for your child to become upset and start crying	☐	☐	☐	☐	☐	☐	☐
4. When your child is crying, he/she usually screams angrily and loudly	☐	☐	☐	☐	☐	☐	☐
5. Your child is very easy to deal with	☐	☐	☐	☐	☐	☐	☐
6. Your child demands an awful lot of attention	☐	☐	☐	☐	☐	☐	☐
7. When your child is left alone, he/she usually plays contentedly on his/her own	☐	☐	☐	☐	☐	☐	☐
8. Your child is so demanding that he/she would pose a major problem for most parents	☐	☐	☐	☐	☐	☐	☐
9. Your child smiles and laughs often	☐	☐	☐	☐	☐	☐	☐
10. Your child is easy to put down and goes to sleep quickly	☐	☐	☐	☐	☐	☐	☐

46. Currently how often does your child usually wake up during the night? *(Enter just one cross.)*

☐ 3 or more times every night
☐ Once or twice every night
☐ A few times a week
☐ Seldom or never

Comments

About yourself

The last time you completed a questionnaire was around week 30 of your pregnancy. The questions we are asking you now are mainly about the period after this up until your child was 6 months old.

Health and use of medication

47. Did you go to your doctor/midwife/health visitor for your own health problems during the first month after the birth?

☐ No
☐ Yes ☐☐ times

48. If yes, what was the reason for this?

☐ Pernineal wound/stitches
☐ Caesarean section wound
☐ Mastitis
☐ Sore nipples
☐ Breastfeeding problems
☐ Other, specify: ____________________

49. When you think back to the time just after the birth, did you feel depressed during that period?

☐ No
☐ Yes, specify how long: ☐☐ weeks

50. Apart from being in hospital for the birth, have you been admitted to hospital since you completed the previous questionnaire?

☐ No
☐ Yes, specify hospital: ____________________

51. Do you have a chronic/long-term illness which has started since you completed the previous questionnaire?

☐ No
☐ Yes, specify: ____________________

52. Overall, how would you describe your physical health at the moment?

☐ Very good
☐ Good
☐ Poor
☐ Very poor

53. Have you had any of the following problems/illnesses since you completed the previous questionnaire? If yes, are you taking or have you taken medication for these problems? *(This includes every type of medication, including natural medicines, taken on both a regular and occasional basis.)* *(Enter a cross in a box for each item.)*

Have you suffered from?				If you have taken medication				
						After the birth		
**Illness / problem**	No	Yes, last part of during pregnancy	Yes, after the birth	Name of medication taken	Last part of this pregnancy	0-3 mth	4-6 mth	Number of days taken in total
1. Sugar in urine	☐	☐	☐	______	☐	☐	☐	☐☐☐
2. Protein in urine	☐	☐	☐	______	☐	☐	☐	☐☐☐
3. High blood pressure . .	☐	☐	☐	______	☐	☐	☐	☐☐☐
4. Swelling (oedema)	☐	☐	☐	______	☐	☐	☐	☐☐☐
5. Cystitis	☐	☐	☐	______	☐	☐	☐	☐☐☐
6. Sluggish bowels/constipation	☐	☐	☐	______	☐	☐	☐	☐☐☐
7. Diarrhoea/vomiting	☐	☐	☐	______	☐	☐	☐	☐☐☐
8. Heartburn/acidity	☐	☐	☐	______	☐	☐	☐	☐☐☐
9. Common cold/influenza	☐	☐	☐	______	☐	☐	☐	☐☐☐
10. Sore throat/sinusitis/earinfection	☐	☐	☐	______	☐	☐	☐	☐☐☐

cont. next page

Have you suffered from?				If you have taken medication				
**Illness / problem**	No	Yes, last part of during pregnancy	Yes, after the birth	Name of medication taken	Last part of this pregnancy	After the birth 0-3 mth	After the birth 4-6 mth	Number of days taken in total
11. Pneumonia/bronchitis . .	☐	☐	☐	________________	☐	☐	☐	☐☐☐
12. Asthma	☐	☐	☐	________________	☐	☐	☐	☐☐☐
13. Hay fever/other allergy.	☐	☐	☐	________________	☐	☐	☐	☐☐☐
14. Headache/other pains .	☐	☐	☐	________________	☐	☐	☐	☐☐☐
15. Vaginitis	☐	☐	☐	________________	☐	☐	☐	☐☐☐
16. Mental health problems	☐	☐	☐	________________	☐	☐	☐	☐☐☐
17. Mastitis.	☐	☐	☐	________________	☐	☐	☐	☐☐☐
18. Fever	☐	☐	☐	________________	☐	☐	☐	☐☐☐
19. Other, specify: ________________	☐	☐	☐	________________	☐	☐	☐	☐☐☐

54. Have you taken medicines other than those mentioned in Question 52? *(For instance, sleeping tablets, sedatives or analgesics.)*

☐ No ☐ Yes

55. If yes, give the name of the medicines and when you took them. *(Include all types of medication, as well as natural medicines, taken both on a regular and occasional basis.)*

**Name of medicine** (e.g. Valium, Rohypnol, Paracetamol)	Last part of pregnancy: Taken medication	Last part of pregnancy: Number of days	0-3 months after the birth: Taken medication	0-3 months after the birth: Number of days	4-6 months after the birth: Taken medication	4-6 months after the birth: Number of days
________________	☐	☐☐☐	☐	☐☐☐	☐	☐☐☐
________________	☐	☐☐☐	☐	☐☐☐	☐	☐☐☐
________________	☐	☐☐☐	☐	☐☐☐	☐	☐☐☐

56. Do you take or have you taken cod liver oil, vitamins or other dietary supplements since the previous questionnaire?

☐ No ☐ Yes

57. If yes, which product, when did you take it and how often? *(One line for each product.)*

**Name of product**	When did you take the product? Last part of pregnancy	0-3 months after the birth	4-6 months after the birth	How often? Taken daily	Taken sometimes
________________	☐	☐	☐	☐	☐
________________	☐	☐	☐	☐	☐
________________	☐	☐	☐	☐	☐

58. Have you experienced any pain in your back or pelvis since you completed the previous questionnaire?

☐ No
☐ Yes

59. If yes, enter a cross to indicate where you have experienced pain, when and how much.

Where was the pain?	Last part of pregnancy: Some pain	Last part of pregnancy: Major pain	0-3 months after the birth: Some pain	0-3 months after the birth: Major pain	4-6 months after the birth: Some pain	4-6 months after the birth: Major pain
Small of the back	☐	☐	☐	☐	☐	☐
One of the pelvic/sacroiliac joints at the back	☐	☐	☐	☐	☐	☐
Both pelvic/sacroiliac joints at the back	☐	☐	☐	☐	☐	☐
Over the coccygeal bone	☐	☐	☐	☐	☐	☐
In the buttocks	☐	☐	☐	☐	☐	☐
Over the pubic bone.	☐	☐	☐	☐	☐	☐
Groin.	☐	☐	☐	☐	☐	☐
Other back pains	☐	☐	☐	☐	☐	☐

60. Currently, do you wake up at night because of pelvic pain?

☐ No, never
☐ Yes, but only sometimes
☐ Yes, often

61. Do you have such problems walking at the moment due to pelvic pain that you have to use a stick or crutches?

☐ No, never
☐ Yes, but not every day
☐ Yes, every day

62. Have you *ever* received treatment for pelvic pain?

☐ No
☐ Yes

63. If yes, enter a cross to indicate the type of treatment and when it was.

	Before this pregnancy	During this pregnancy	After this birth
Physiotherapy	☐	☐	☐
Chiropractic	☐	☐	☐
Medication	☐	☐	☐
Other, specify: ___________________	☐	☐	☐

64, How long was it before you resumed sexual intercour-se after the birth?

☐☐ weeks

☐ Have not had sexual intercourse

65. Do you have any of the following problems at the moment; if so, how often and to what extent? *(Enter a cross in a box for each item.)*

Problem	How often do you have these problems?: Never	1-4 times a month	1-6 times a week	Once a day	More than Once a day	How much at a time?: Drops	Large amounts
Incontinence when coughing, sneezing or laughing . . .	☐	☐	☐	☐	☐	☐	☐
Incontinence during physical activity (running/jumping)	☐	☐	☐	☐	☐	☐	☐
Incontinence with a strong need to urinate	☐	☐	☐	☐	☐	☐	☐
Problems retaining faeces.	☐	☐	☐	☐	☐	☐	☐
Problems with flatulence	☐	☐	☐	☐	☐	☐	☐

66. How many times did you go for an ultrasound scan during your pregnancy?

☐☐ times

67. Was everything OK with the ultrasound scan(s)?

☐ Yes
☐ No

68. If no, what was the problem?

☐ The baby was not growing enough.
☐ Suspected malformation,describe:

____

☐ Other, specify: _______________________________________

69. How much did you weigh at the end of your pregnancy and how much do you weigh now?

At end of pregnancy [ ][ ][ ],[ ] kg

Now [ ][ ][ ],[ ] kg

70. Were you completely or partly on sick leave after week 30 of your pregnancy? *(Don't include maternity leave)*

- ☐ No
- ☐ Yes,partly on sick leave
- ☐ Yes,completely on sick leave

71. If you were on sick leave after week 30 of your pregnancy, complete the table below with a line for each time you were on sick leave. Give the reason and enter a cross indicating which weeks of your pregnancy you were on sick leave. Specify how many days and what percentage of the period you were on sick leave each time.

Reason for sick leave:	Was on sick leave during pregnancy weeks 30-33	34-37	38+	Number of days	% sick leave
Example: pelvic girdle pains	☐	☒	☐	1 0	5 0
______	☐	☐	☐		
______	☐	☐	☐		
______	☐	☐	☐		

Finances – lifestyle

72. Would your current financial situation allow you to cope with an unexpected bill of NOK 10,000 for a dental visit or a repair, for a instance?

- ☐ No
- ☐ Yes
- ☐ Don't know

73. Have you found it difficult sometimes during the last six month to cope with running expemces for food, transport, rent etc.?

- ☐ No, never
- ☐ Yes, but infrequently
- ☐ Yes, sometimes
- ☐ Yes, often

74. Are there pets in the child's home?

- ☐ No
- ☐ Yes

75. If yes, which type(s)? *(You can enter a cross in more than one box.)*

- ☐ Dog
- ☐ Cat
- ☐ Guinea pig, rabbit, mouse, rat, etc.
- ☐ Budgie, other type of bird
- ☐ Other type of animal:

______

76. Do you have heating based on electrical heating cables under the floor in rooms where you child is? (Do not include waterborne heating)

- ☐ No
- ☐ Yes

77. If yes, in which rooms? *(You can enter a cross in more than one box.)*

- ☐ Living room
- ☐ Kitchen
- ☐ Child's room
- ☐ Bedroom
- ☐ Hall
- ☐ Bathroom
- ☐ Other rooms

78. How often do you exercise these muscle groups at home or at the gym at present? *(Enter a cross in a box for each item.)*

	Never	1-3 times times a month	Once a week	Twice a week	Three times or more a week
Stomach muscles	☐	☐	☐	☐	☐
Back muscles	☐	☐	☐	☐	☐
Pelvic floor muscles (muscles around the vagina, urethra, rectum)	☐	☐	☐	☐	☐

79. How often are you physically active at present? *(Enter a cross in a box for each item.)*

	Never	1-3 times a month	Once a week	Twice a week	Three times or more a week
1 Didn't smoke	☐	☐	☐	☐	☐
2 Brisk walking	☐	☐	☐	☐	☐
3 Running/jogging/orienteering	☐	☐	☐	☐	☐
4 Cycling	☐	☐	☐	☐	☐
5 Training studio/weight training	☐	☐	☐	☐	☐
6 Special gymnastics/aerobics for pregnant women	☐	☐	☐	☐	☐
7 Aerobics/gymnastics/dancing without running and jumping	☐	☐	☐	☐	☐
8 Aerobics/gymnastics/dancing with running and jumping	☐	☐	☐	☐	☐
9 Dancing (swing, rock, folk)	☐	☐	☐	☐	☐
10 Skiing	☐	☐	☐	☐	☐
11 Ball sport	☐	☐	☐	☐	☐
12 Swimming	☐	☐	☐	☐	☐
13 Riding	☐	☐	☐	☐	☐
14 Other	☐	☐	☐	☐	☐

80. Currently how often are you physically active (during your spare time or at work) that you get out of breath or sweat?

	Spare time	At work
Never	☐	☐
Less than once a week	☐	☐
Once a week	☐	☐
Twice a week	☐	☐
3-4 times a week	☐	☐
5 times or more a week	☐	☐

81. What were your and your partner/husband's smoking habits during the last 3 months of your pregnancy and in the period after the birth? *(Enter a cross in a box for each period.)*

	Yourself			Your partner/husband		
	Last 3 mths during pregnancy	0-3 mths after birth	4-6 mths after birth	Last 3 mths during pregnancy	0-3 mths after birth	4-6 mths after birth
Didn't smoke	☐	☐	☐	☐	☐	☐
Smoked sometimes	☐	☐	☐	☐	☐	☐
Smoked every day	☐	☐	☐	☐	☐	☐
If every day, number of cigarettes per day	☐☐	☐☐	☐☐	☐☐	☐☐	☐☐
If sometimes, number of cigarettes per week	☐☐	☐☐	☐☐	☐☐	☐☐	☐☐

82. Is your child ever present in a room where someone smokes?

☐ No
☐ Yes, sometimes
☐ Yes, several times a week
☐ Yes, every day

If every day, number of hours ☐☐

83. Did you take any of the following substances during the last 3 months of your pregnancy and after the birth?
(Enter a cross in a box for each item.)

	No	Yes, last 3 month of pregnancy	Yes after birth
Hanish	☐	☐	☐
Amphetamines	☐	☐	☐
Ecstasy	☐	☐	☐
Cocaine	☐	☐	☐
Heroin	☐	☐	☐
Other, specify: ______	☐	☐	☐

84. Have you taken any of the following substances during the last 3 months of your pregnancy and after the birth? *(Enter a cross in a box for each item.)*

	No	Yes, last 3 months of pregnancy	Yes, after birth
Anabolic steroids	☐	☐	☐
Testosterone preparations	☐	☐	☐
Growth hormone (e.g. genotropin/somatropin)	☐	☐	☐

85. How often did you drink alcohol during the last 3 months of your pregnancy and how often do you drink now? *(Enter a cross in a box for each period.)*

	Last 3 months of pregnancy	After the birth	
		0-3 months	4-6 months
Roughly 6-7 times a week	☐	☐	☐
Roughly 4-5 times a week	☐	☐	☐
Roughly 2-3 times a week	☐	☐	☐
Roughly once a week	☐	☐	☐
Roughly 1-3 times a month	☐	☐	☐
Less often than once a month	☐	☐	☐
Never	☐	☐	☐

Alcohol units
In order compare different types of alcohol, we ask for the number of alcohol units (= 1.5 cl of pure alcohol). In practice, this means the following:

1 glass (1/3 litre) of beer	= 1 alcohol unit
1 wine glass of red or white wine	= 1 alcohol unit
1 sherryglass of sherry	= 1 alcohol unit
1 brandy glass of spirits or liquer	= 1 alcohol unit
1 bottle of alcopop/cider	= 1 alcohol unit

86. How many units of alcohol do you usually drink when you consume alcohol (complete both for the last 3 months of your pregnancy and afterwords)? *(See explanation about alcohol units.) (Enter a cross in a box for each period.)*

Number of alcohol units	Last 3 months of pregnancy	After the birth	
		0-3 months	4-6 months
10 or more	☐	☐	☐
7-9	☐	☐	☐
5-6	☐	☐	☐
3-4	☐	☐	☐
1-2	☐	☐	☐
Less than 1	☐	☐	☐

A little more about yourself and how you are keeping now

87. Do you have a boyfriend/ husband/partner?

☐ Yes
☐ No

88. If yes, to what extent do you agree with the following descriptions? *(Enter just one cross in a box for each item.)*

	Totally agree	Agree	Slightly agree	Slightly disagree	Disagree	Totally disagree
My husband/partner and I have a close relationship	☐	☐	☐	☐	☐	☐
My partner and I have problems in our relationship	☐	☐	☐	☐	☐	☐
I am very happy in my relationship	☐	☐	☐	☐	☐	☐
My partner is usually understanding	☐	☐	☐	☐	☐	☐
I often think about ending our relationship	☐	☐	☐	☐	☐	☐
I am satisfied with my relationship with my partner	☐	☐	☐	☐	☐	☐
We often disagree about important decisions	☐	☐	☐	☐	☐	☐
I have been lucky in my choice of partner	☐	☐	☐	☐	☐	☐
We agree on how children should be raised	☐	☐	☐	☐	☐	☐
I think my partner is satisfied with our relationship	☐	☐	☐	☐	☐	☐

89. In your daily life, how often do you *(Enter just one cross in a box for each item.)*

	Seldom never	Fairly seldom	A few times	Often	Very often
Feel pleased about something	☐	☐	☐	☐	☐
Feel happy	☐	☐	☐	☐	☐
Feel joyful, as though everything is going your way	☐	☐	☐	☐	☐
Feel that you will scream at someone or hit something	☐	☐	☐	☐	☐
Feel angry, irritated or annoyed	☐	☐	☐	☐	☐
Feel mad at somebody	☐	☐	☐	☐	☐

90. Indicate with a cross whether you agree or disagree with the following statements.
(Enter just one cross in a box for each item.)

	Totally disagree	Disagree	Slightly disagree	Neither agree or disagree	Slightly agree	Agree	Totally agree
My life is largely what I wanted it to be	☐	☐	☐	☐	☐	☐	☐
My life is very good	☐	☐	☐	☐	☐	☐	☐
I am satisfied with my life	☐	☐	☐	☐	☐	☐	☐
I have achieved so far what is important for me in my life	☐	☐	☐	☐	☐	☐	☐
If I could start all over, there is very little I would do differently	☐	☐	☐	☐	☐	☐	☐

91. Have you experienced any of the following situations since the previous questionnaire? If yes, how painful or difficult was this for you? *(Enter a cross in a box for each item.)*

			If yes		
	No	Yes	Not so bad	Painful/ difficult	Very painful/ difficult
Have you had problems at work or where you study?	☐	☐	☐	☐	☐
Have you had financial problems?	☐	☐	☐	☐	☐
Have you been divorced, separated or ended your relationship with your partner?	☐	☐	☐	☐	☐
Have you had problems or conflicts with family, friends or neighbours?	☐	☐	☐	☐	☐
Have you been seriously worried that there is something wrong with your child?	☐	☐	☐	☐	☐
Have you been seriously ill or injured?	☐	☐	☐	☐	☐
Has anyone close to you been seriously ill or injured?	☐	☐	☐	☐	☐
Have you been involved in a serious accident, fire or robbery?	☐	☐	☐	☐	☐
Have you lost someone close to you?	☐	☐	☐	☐	☐
Have you been pressurized into having sexual intercourse?	☐	☐	☐	☐	☐
Other	☐	☐	☐	☐	☐

92. Have you experienced any of the following feelings during the last week? *(Enter just one cross in a box for each item.)*

	Yes, almost all the time	Yes, now and then	Not very often	No, never
Really reproached yourself when something went wrong	☐	☐	☐	☐
Have been anxious or worried for no reason	☐	☐	☐	☐
Have been afraid or panicked for no reason	☐	☐	☐	☐
Have been so unhappy that you've had problems sleeping	☐	☐	☐	☐
Felt down or unhappy	☐	☐	☐	☐
Have been so unhappy that you've cried	☐	☐	☐	☐

93. How do you feel about yourself? *(Enter just one cross in a box for each item.)*

	Totally agree	Agree	Disagree	Totally disagree
I have a positive attitude towards myself	☐	☐	☐	☐
I feel completely useless at times	☐	☐	☐	☐
I feel that I do not have much to be proud about	☐	☐	☐	☐
I feel that I am a valuable person, as good as anyone else	☐	☐	☐	☐

94. Have you been bothered by any of the following feelings during the past 2 weeks? *(Enter just one cross in a box for each item.)*

	Not bothered	A little bothered	Quite bothered	Very bothered
Feeling fearful	☐	☐	☐	☐
Nervousness or shakiness inside	☐	☐	☐	☐
Feeling hopeless about the future	☐	☐	☐	☐
Feeling blue	☐	☐	☐	☐
Worrying too much about things	☐	☐	☐	☐
Feeling everything is an effort	☐	☐	☐	☐
Feeling tense or keyed up	☐	☐	☐	☐
Suddenly scared for no reason	☐	☐	☐	☐

Thank you very much for your help!

Insert the completed questionnaire in the stamped addressed envelope.
